# Recent advances in theranostic nanomaterials for overcoming traumatic brain injury

**DOI:** 10.1186/s12951-025-03685-4

**Published:** 2025-10-29

**Authors:** Nam Cheol Hwang, Dong Min Lim, Tae Sik Goh, Jung Mo Kang, Jaehoon Kim, Shin Kim, Yun Hak Kim, Dokyoung Kim

**Affiliations:** 1 Department of Biomedical Science, Graduate School, Kyung Hee University, Seoul, 02447 Republic of Korea; 2 Interdisciplinary Program of Genomic Data Science, Pusan National University, Yangsan, 50612 Republic of Korea; 3 Biomedical Research Institute, Pusan National University Hospital, Busan, 49241 Republic of Korea; 4 Department of Orthopaedic Surgery, Pusan National University School of Medicine, Yangsan, 50612 Republic of Korea; 5 College of Medicine, Kyung Hee University, Seoul, 02447 Republic of Korea; 6 Department of Immunology, School of Medicine, Keimyung University, Dalseo-gu, Daegu, 42601 Republic of Korea; 7 Institute of Medical Science, Keimyung University, Dalseo-gu, Daegu, 42601 Republic of Korea; 8 Institute for Cancer Research, Keimyung University Dongsan Medical Center, Dalseo-gu, Daegu, 42601 Republic of Korea; 9 Department of Anatomy, School of Medicine, Pusan National University, Yangsan, 50612 Republic of Korea; 10 Department of Biomedical Informatics, School of Medicine, Pusan National University, Yangsan, 50612 Republic of Korea; 11 Research Institute for Convergence of Biomedical Science and Technology, Pusan National University Yangsan Hospital, Yangsan, 50612 Republic of Korea; 12 Department of Precision Medicine, Graduate School, Kyung Hee University, Seoul, 02447 Republic of Korea; 13 Department of Anatomy and Neurobiology, College of Medicine, Kyung Hee University, Seoul, 02447 Republic of Korea; 14 Department of Converging Science and Technology, Kyung Hee University, Seoul, 02447 Republic of Korea; 15 Medical Research Center for Bioreaction to Reactive Oxygen Species and Biomedical Science Institute, Core Research Institute (CRI), Kyung Hee University, Seoul, 02447 Republic of Korea

**Keywords:** Brain, Nanotherapeutics, Drug delivery, Translational research, Biomedical engineering

## Abstract

Traumatic brain injury (TBI) is a major global health challenge characterized by complex secondary injury mechanisms involving oxidative stress, inflammation, and blood-brain barrier (BBB) disruption. Traditional biosensing and therapy strategies often face limitations due to ineffective biomarker detection and poor drug targeting at the injury site. Recently, theranostic nanomaterials have emerged as a promising solution, integrating diagnostic and therapeutic functionalities within a single nanoscale platform. This review explores the latest advances in nanotherapeutics and nanosensors for TBI management. The first section summarizes various nanotherapeutic approaches, including PEGylated-polystyrene nanoparticles, porous silicon nanoparticles, carbon dot nanoparticles, dendrimer nanoparticles, lipid nanoparticles (LNPs), and siRNA-based nanoparticles, all of which have demonstrated enhanced neuroprotection and targeted drug delivery in TBIs. Notably, LNPs exhibit further optimized biocompatibility and therapeutic efficacy, while carbon dot nanoparticles function as nanozymes to combat oxidative stress, thereby mitigating neuronal damage. The second section focuses on nanosensors for TBIs, including peptide-based nanosensors, ECM-targeted nanosensors, and biomarker-responsive platforms capable of real-time diagnosis and monitoring of TBI progression. Additionally, the use of polymeric and fibrinogen-based nanosensors is discussed as advanced strategies to improve precision detection and therapeutic control. This review provides a comprehensive overview of theranostic nanomaterials for TBIs, highlighting their transformative potential in diagnosis and targeted treatment, while addressing key translational challenges to clinical application.

**Graphical Abstract:**

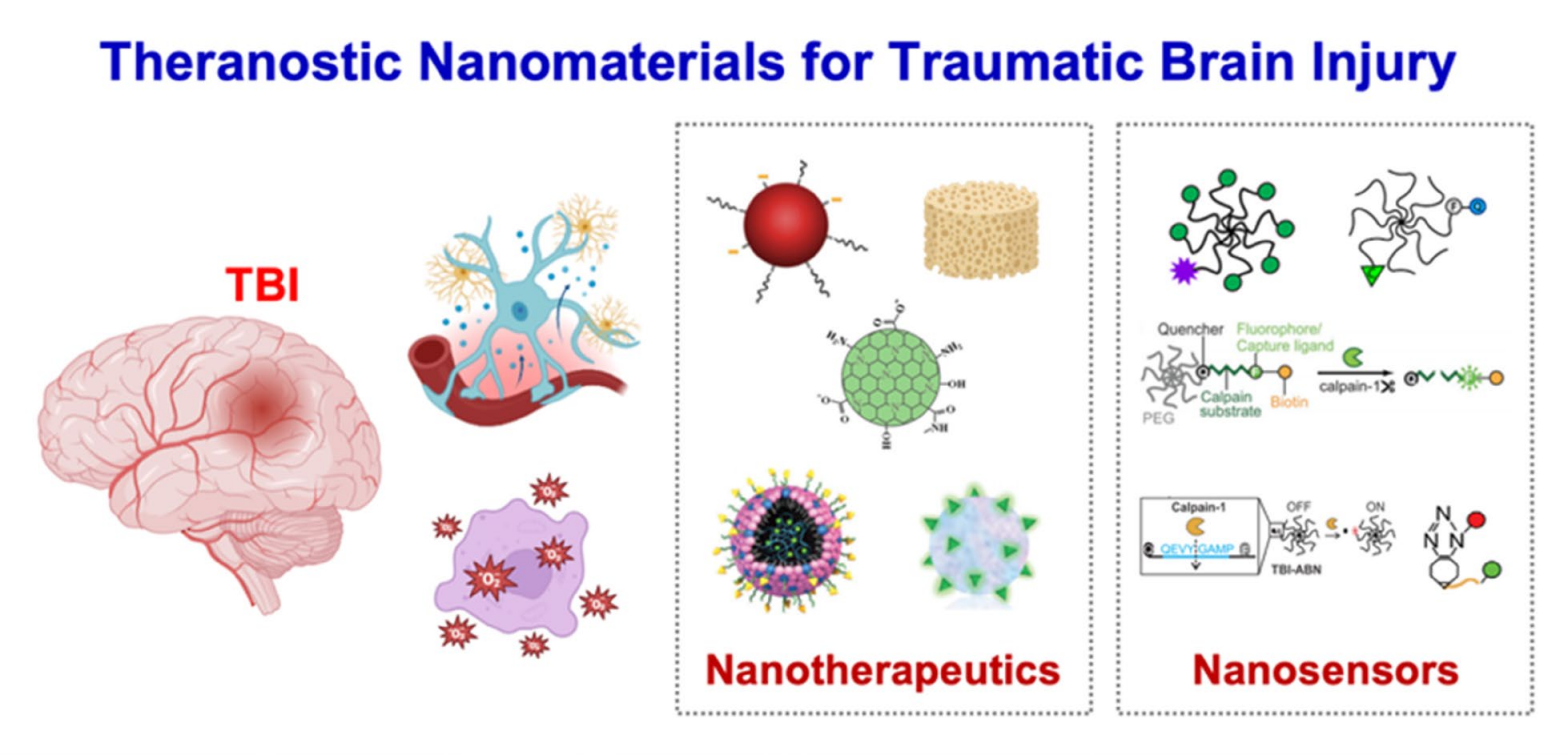

**Supplementary Information:**

The online version contains supplementary material available at 10.1186/s12951-025-03685-4.

## Introduction

Traumatic brain injury (TBI) represents a significant global public health concern, characterized by complex pathophysiological mechanisms and a wide spectrum of clinical manifestations [1–3]. TBI results from abrupt external forces such as falls, vehicular collisions, sports-related impacts, or blasts, leading to immediate mechanical damage to brain tissue. This primary injury is often compounded by secondary injury mechanisms that include excitotoxicity, neuroinflammation, oxidative stress, and disruption of the blood-brain barrier (BBB) [4–6]. These cascading events contribute to long-term neurological deficits, such as cognitive impairment, motor dysfunction,

and behavioural disturbances, ultimately diminishing quality of life and imposing substantial socioeconomic burdens. Despite advancements in neuroimaging and clinical management, early diagnosis and effective treatment remain challenging. Conventional diagnostic tools, though useful, often lack the sensitivity to detect subtle or progressive changes in brain tissue. Moreover, traditional therapeutic approaches are often inadequate due to the heterogeneous nature of TBI pathology and the difficulty in achieving targeted, controlled drug delivery [7, 8]. This is further complicated by the protective but restrictive nature of the BBB, which significantly limits the efficient penetration of many therapeutic agents into the brain [9, 10].

In this context, theranostic nanomaterials have emerged as a promising strategy to revolutionize the management of TBI. Theranostics, an integrated approach combining diagnostic and therapeutic functionalities within a single nanoscale platform, offers a dual advantage. These nanomaterials can be engineered to enhance imaging contrast, enabling precise localization and real-time monitoring of brain lesions [11, 12]. In addition, they facilitate the targeted delivery of neuroprotective agents directly to the affected regions, thereby minimizing systemic side effects and maximizing therapeutic efficacy. The unique physicochemical properties of nanomaterials, such as high surface-to-volume ratio, tunable size, and modifiable surface chemistry, allow them to traverse the BBB and deliver therapeutic payloads in a controlled manner [13, 14]. Recent advancements in this field of TBI research have highlighted the potential of various nanoplatforms, including liposomes, polymeric nanoparticles, hybrid nanostructures, and emerging systems in nanotherapeutics and nanosensors, for achieving both early diagnosis and effective treatment. Building on recent advances, PEGylated nanozymes targeting oxidative stress, glucose-mimicking nanovesicles co-delivering siRNA and 2-deoxyglucose, glutamate-targeted electrochemical sensors enhanced with electrodeposited nanoplatinum, LDH-sensing microfluidic brain-on-chip models, and label-free electrochemical impedance spectroscopy (EIS)-based diagnostic platforms collectively represent recent efforts toward multifunctional and integrated strategies in TBI management. However, recent studies in this area remain relatively limited [15–19]. These systems are designed to respond to specific pathological cues within the injured brain, such as pH changes, enzyme activity, or elevated levels of reactive oxygen species (ROS), thereby enabling stimuli-responsive, controlled drug release. Moreover, the incorporation of imaging agents, such as magnetic resonance imaging (MRI) contrast materials or fluorescent probes, into these nanoplatforms allows clinicians to monitor biodistribution, accumulation, and therapeutic response in real-time [20, 21]. The convergence of nanotechnology and neuroscience has opened new avenues for personalized medicine in TBI management. By providing a means for simultaneous diagnosis and therapy, theranostic nanomaterials hold the promise of improving clinical outcomes, while deepening our understanding of the underlying mechanisms of brain injury. Continued interdisciplinary research is essential to further optimize these platforms, address potential toxicity issues, and facilitate their translation from bench to bedside. Ultimately, the development of advanced theranostic nanomaterials represents a significant step toward overcoming the limitations of current TBI monitoring and treatments, paving the way for more effective and individualized therapeutic strategies [22, 23].

In this comprehensive review, we systemically summarized recent advances in theranostic nanomaterials, highlighting novel approaches to material design and practical applications. The review is organized into five main sections: (1) Introduction, (2) Brain Injury: Causes, Mechanisms of injury Progression, Clinical therapy by access, Nano-driven approaches for TBI treatment, (3) Nanotherapeutics for TBIs: six subcategories based on material types, (4) Nanosensors for TBIs: five subcategories based on material classes, and (5) Conclusion and Perspective. From the current standpoint, nanomaterial-based strategies are one of the most promising approaches for overcoming the challenges associated with TBIs. The materials discussed in this manuscript include nanoparticles, polymers, target peptide nanoparticles, siRNA nanoparticles, lipid nanoparticles (LNPs), and nanozymes. In particular, the final subcategory, click chemistry-based nanoplatforms, illustrates the potential of chemical modification capabilities and the use of click chemistry as an emerging tool for achieving therapeutic goals in TBI management. We hope that the summary, insights, and perspectives in this review will contribute to future research and development in both basic research and translational medicine.

## Brain injury

### Causes

**Fig. 1:**
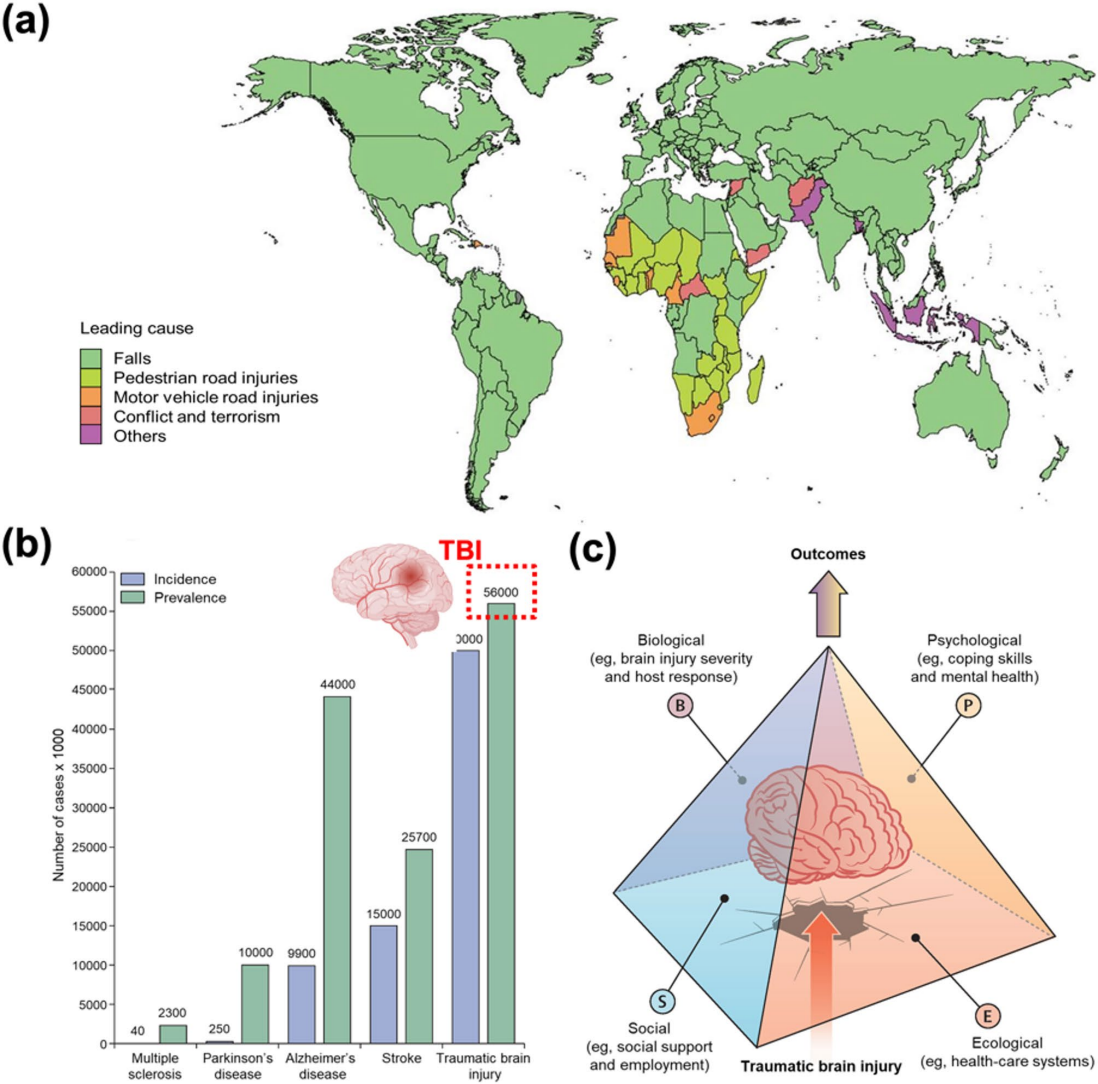
Overview of TBI occurrences. (**a**) Global distribution of TBI etiologies (2019 GBD study). Copyright (2023) BMJ. (**b**) Annual incidence of TBIs and associated economic impact. (**c**) Influence of individual and environmental factors on TBI outcomes: the bio-psycho-socio-ecological model. Copyright (2022) Elsevier

The 2019 Global Burden of Disease (GBD) study highlights the diverse etiologies of TBIs, identifying falls as the leading cause in 74% of countries and territories, followed by pedestrian road injuries (14%), motor vehicle road injuries (5%), and conflict and terrorism (2%) (Fig. 1a) [24, 25]. Among these, vehicular accidents are significant, often involving high-impact collisions that result in concussions and diffuse axonal injuries due to rapid deceleration [26, 27]. Similarly, high-contact sports such as football, boxing, and rugby are associated with repetitive head trauma, which has been increasingly linked to the development of chronic traumatic encephalopathy (CTE), a progressive neurodegenerative disorder marked by cognitive decline, mood disturbances, and motor dysfunction [28–31]. Falls, especially among the elderly, represent another major contributor to TBIs. In this demographic, age-related fragility and comorbidities exacerbate injury severity, prolong recovery, and increase morbidity [32–34]. Military-related TBIs, though less common in the general population, pose a significant concern for soldiers, especially those returning from deployment in conflict zones such as Iraq and Afghanistan [35, 36]. Exposure to blast waves from improvised explosive devices (IEDs), artillery shells, and shrapnel can cause primary injuries such as intracranial pressure fluctuations, skull deformation, vascular damage, and secondary penetrating injuries. Both injury types can compromise the integrity of the BBB, increasing the risk of chronic neurological conditions including cognitive impairment, post-traumatic stress disorder (PTSD), and CTE. Moreover, repeated low-level blast exposure has been associated with neuroinflammation, tauopathy, heightened neuronal excitability, and non-convulsive seizures in animal models [37–42]. These multifactorial injury pathways necessitate a comprehensive understanding of TBI pathophysiology to develop targeted therapeutic strategies aimed at mitigating long-term neurological and psychiatric conditions, specifically in military populations.

According to the 2017 commission, TBIs were projected to remain among the top three causes of injury-related mortality and disability through 2030. Each year, an estimated 50–60 million individuals sustain a TBI, contributing to a substantial global economic burden of approximately US$400 billion (Fig. 1b). Among neurological disorders, TBIs exhibit the highest incidence, underscoring their profound impact on public health. Furthermore, the impacts of TBIs are increasingly shaped by both individual and environmental factors, including age, pre-injury mental health status, coping capacity, and broader life course determinants such as litigation and access to healthcare (Fig. 1c). These diverse etiologies highlight the multifactorial nature of TBIs, highlighting the need for personalized therapeutic strategies that address specific pathophysiological mechanisms and demographic variables associated with each cause [8]. In this context, nanoscience has emerged as a promising avenue for TBI treatment, offering innovative strategies for neuroprotection, neural repair, and targeted drug delivery to injured regions of the brain [43, 44]. Advancements in nanotechnology may pave the way for more effective therapeutic interventions aimed at mitigating the long-term consequences of TBIs.

### Mechanisms of injury progress

TBIs begin with a primary injury, characterized by immediate mechanical damage that shears neurons, glial cells, and blood vessels. While this initial insult is irreversible and can only be prevented through proactive safety measures, it triggers a complex cascade of secondary injury mechanisms. This secondary phase progresses over time and is driven by oxidative stress, excitotoxicity, mitochondrial dysfunction, and neuroinflammation—factors that disrupt cellular homeostasis and contribute to progressive neuronal cell death. Moreover, the compromise of vascular integrity further exacerbates injury progression, and these pathological processes can persist well beyond the initial trauma, leading to sustained chronic neurodegeneration and a marked decline in quality of life [45–47].

**Fig. 2:**
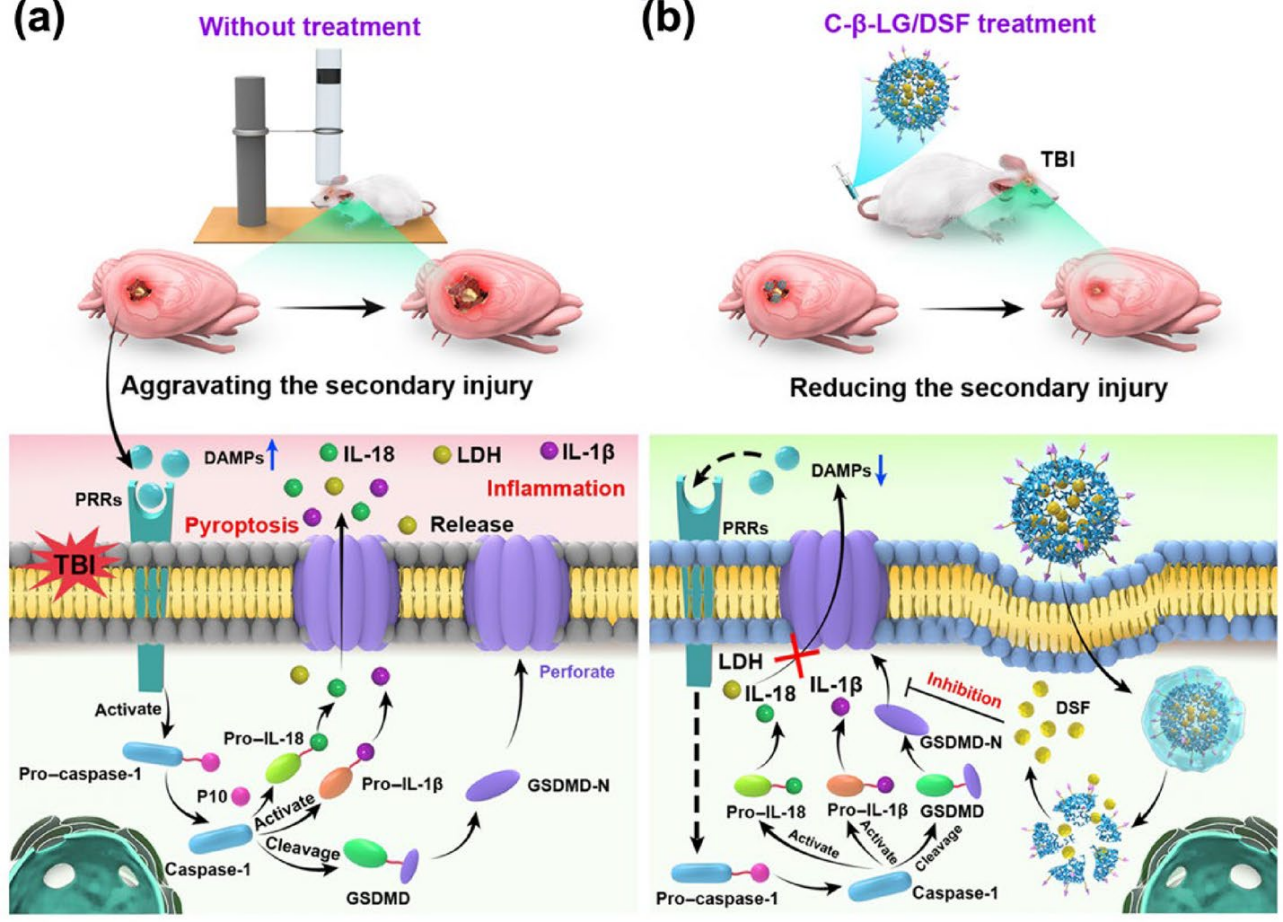
Pathophysiological mechanisms in TBIs. (**a**) Post-TBI cascade: DAMP-induced inflammasome activation via PRRs, triggering pyroptosis and the release of inflammatory cytokines. (**b**) C-β-LG/DSF treatment inhibits the formation of GSDMD-N pores, suppressing pyroptosis and reducing cytokine release. Copyright (2024) American Association for the Advancement of Science

In the absence of therapeutic intervention, TBIs induce the release of damage-associated molecular patterns (DAMPs), which activate pattern recognition receptors (PRRs) and promote inflammasome assembly (Fig. 2a). This cascade ultimately triggers pyroptosis through the formation of gasdermin D (GSDMD-N) pores, resulting in the release of pro-inflammatory cytokines such as IL-1β and IL-18. The resulting inflammatory response amplifies neuroinflammation and further contributes to secondary brain injury. Since the primary insult, which involves direct mechanical damage, cannot be therapeutically reversed, current treatment strategies focus on limiting secondary injury (i.e., delayed non-mechanical damage). This phase is affected by alterations in cerebral blood flow (including both hypo- and hyperperfusion), impairment of cerebrovascular autoregulation, cerebral metabolic dysfunction, and insufficient oxygenation. Secondary injury unfolds over minutes to days following the initial event and presents a critical opportunity for intervention. Therefore, effective intervention during this stage aims to mitigate secondary damage, which is exacerbated by alterations in cerebral blood flow, impaired cerebrovascular autoregulation, metabolic dysfunction, and inadequate oxygen delivery to brain tissue [47–50].

Recent advances have underscored the therapeutic potential of nanoparticle-based strategies for TBI treatment. Notably, Zhang and co-workers demonstrated that targeting pyroptosis with nanoparticles effectively attenuates neuroinflammatory responses, thereby preventing secondary damage in a TBI model [51]. This approach highlights the promise of nanoparticle-mediated interventions in disrupting pathological cascades and enhancing clinical outcomes in TBI management. One such intervention, post-TBI administration of C-β-LG/DSF, disrupts this deleterious cascade by inhibiting the formation of GSDMD-N pores, thereby reducing the release of inflammatory cytokines (Fig. 2b). This targeted therapeutic strategy, capitalizing on recent advancements in nanoparticle engineering and material science, has demonstrated considerable efficacy in mitigating neuroinflammation and attenuating secondary brain injury. These findings emphasize its promising potential for the development of next-generation treatments for TBIs.

Despite these promising advances, significant obstacles continue to hinder the clinical translation of nanoparticle-based therapies for TBI. These challenges often arise from insufficient drug targeting and retention at the injury site, as well as an incomplete understanding of the complex and heterogeneous pathophysiology underlying TBI. Moreover, critical questions persist regarding the long-term safety, optimal dosing regimens, and potential off-target effects of these nanotechnologies, all of which must be rigorously addressed before widespread clinical adoption [52, 53]. Nonetheless, functionalized nanoparticles represent a compelling therapeutic strategy by safeguarding pharmacological payloads, improving targeted delivery, and working synergistically with biomaterial scaffolds to enhance therapeutic efficacy. Ultimately, the successful clinical implementation of nanotechnology in neurology and neurosurgery will rely on coordinated advancements in materials science, more comprehensive insights into nervous system biology, and the rational integration of nanoengineered platforms [54]. Collectively, these developments are anticipated to drive the emergence of targeted interventions that foster axonal regeneration and neural repair in the context of brain injury.

### Clinical therapy by access

**Fig. 3:**
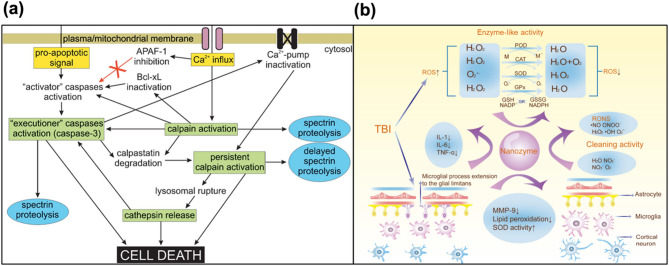
Calpain-caspase activation and nanozyme-driven ROS scavenging. (**a**) Schematic illustration of Ca²⁺-induced calpain activation and its interplay with caspases. Copyright (2005) Springer. **(b)** Schematic diagram showing how nanozymes scavenge ROS, enhance peroxide dismutase activity and reduce lipid peroxidation, pro-inflammatory cytokine production, and MMP-9 expression. Copyright (2024) American Chemical Society

Elevated intra-axonal Ca^2+^ levels activate the protease calpain, triggering calpain-mediated proteolysis of cytoskeletal proteins and leading to irreversible axonal damage. Numerous studies have investigated the use of calpain inhibitors to mitigate neurodegeneration in TBIs [55–61]. Calpain-1 is a calcium-dependent protease abundantly expressed in neurons, glia, and endothelial cells within the central nervous system. In the context of TBI, pathological elevations in intracellular calcium lead to sustained activation of calpain-1. This aberrant activation initiates the proteolytic cleavage of key structural proteins, resulting in the production of breakdown products such as myelin basic protein, neurofilaments, and αII-spectrin. Notably, these proteolytic fragments are actively being investigated as potential biomarkers for TBI, as their concentrations correlate with injury severity and patient prognosis. Elevated levels of spectrin breakdown products generated by calpain- and caspase-mediated proteolysis have been consistently detected in acute neuronal injury. These findings underscore the value of spectrin breakdown products as diagnostic and prognostic biomarkers in this context. Collectively, these data establish the central role of calpain-1 in the molecular sequelae of TBI and support the utility of both its enzymatic activity and its downstream breakdown products as promising diagnostic and prognostic indicators [56, 62–68]. In addition to its function in proteolytic cleavage, calpain-1 activation has also been implicated in mediating various forms of cell death following neural injury. Specifically, post-ischemic neuronal death may involve both necrotic and apoptotic mechanisms at the single-cell level, a process that is driven in part by the upstream role of calpain in activating caspases. Caspase-3, in turn, degrades calpastatin and thereby indirectly enhances calpain activity (Fig. 3a) [63]. However, earlier studies reported only limited success in using calpain inhibitors to prevent acute hippocampal neurodegeneration [60]. More recent therapeutic approaches are aimed at improving their efficacy. One notable strategy is the development of activity-based nanotheranostic platforms, as demonstrated by Madias et al. [69]. This platform not only senses calpain activity but also suppresses it, thereby reducing calpain-mediated damage. Beyond the excessive influx of calcium, ROS represent another major pathological factor in TBIs. Following the trauma, ROS are generated from various sources, including enzymatic processes, activated neutrophils, excitatory pathways, and dysfunctional mitochondria [70]. In particular, mitochondrial dysfunction leads to oxidative stress, energy failure, and accelerated neuronal damage [71]. This oxidative stress may result from either excessive mitochondrial ROS production or from compromised mitochondrial antioxidant systems. The incomplete reduction of molecular oxygen during oxidative phosphorylation generates ROS, which in turn damages the inner mitochondrial membrane by oxidizing its phospholipids and integral proteins. This reduces membrane fluidity and impairs the function of soluble electron carriers. Protein oxidation also disturbs three-dimensional conformation, promoting aggregation, fragmentation, and susceptibility to proteolysis, ultimately leading to diminishing function [72–75].

The cerebroprotective effects observed in TBIs are largely attributed to the modulation of ROS-mediated pathways. Therapeutic strategies that target ROS can attenuate oxidative stress by scavenging free radicals and enhancing endogenous peroxide dismutase activity. These strategies diminish lipid peroxidation and preserve neuronal membrane integrity. Additionally, by suppressing the release of pro-inflammatory cytokines and downregulating matrix metalloproteinase-9 (MMP-9) expression, these interventions effectively dampen the neuroinflammatory response associated with TBIs. Collectively, these mechanisms underscore the potential of ROS-targeted therapies in ameliorating secondary brain injury post-trauma (Fig. 3b) [76].

**Fig. 4:**
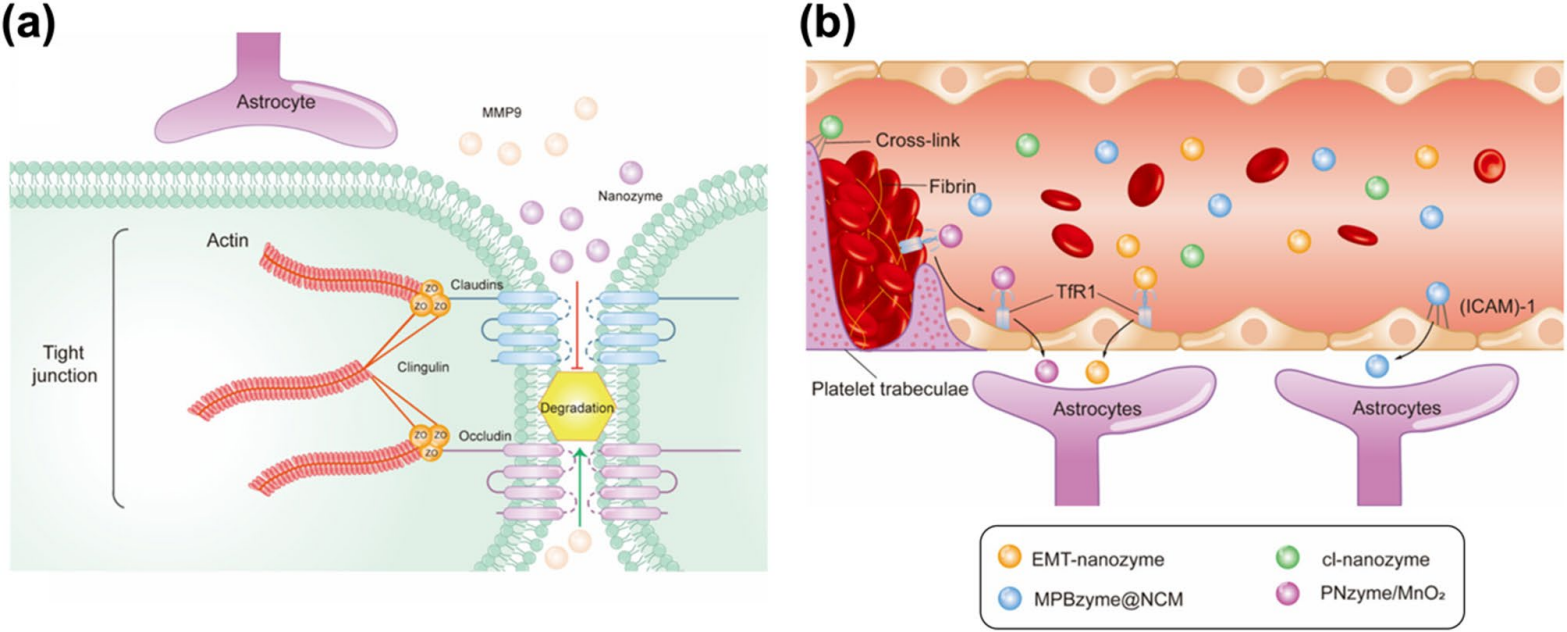
Nanoenzyme-mediated BBB repair and penetration mechanisms. (**a**) Schematic diagram illustrating the restoration of tight junction proteins by nanozymes, which protect the BBB integrity by scavenging ROS, inhibiting glial activation, and reducing MMP-9 production. (**b**) Schematic illustration showing that nanozymes traverse the BBB through three separate pathways: cl-nanozyme through thrombus incorporation; MPBzyme@NCM via ICAM-1-mediated transcytosis; and PNzyme/MnO_2_/EMT-nanozyme via Tf-mediated endocytosis. Copyright (2024) American Chemical Society

Another primary challenge in treating TBIs is the effective delivery of therapeutic agents to the brain, a process significantly hindered by the restrictive properties of the BBB. Disruption of the BBB following a TBI can persist for several days, and in some cases, even for years after the initial injury. Furthermore, the prolonged breakdown of the BBB contributes substantially to the onset of long-term TBI-related complications, including Alzheimer’s disease, cognitive dysfunction, psychological disorders, and epilepsy [77]. BBB disruption is a well-established feature of TBIs and enables the passive accumulation of nanomaterials in perilesional brain tissue, as demonstrated in multiple studies [43, 58, 78–82]. At the molecular level, the BBB integrity is compromised through the degradation of tight junction proteins, such as Zonula Occludens-1 and Claudin-5, by MMP-9 secreted from activated microglia and astrocytes. Nanozymes can help restore these junctional proteins and safeguard the barrier by scavenging ROS and suppressing glial activation, thereby reducing MMP-9 production (Fig. 4a) [76]. This vascular damage enables nanoparticles to penetrate the injured brain via passive diffusion across the dysregulated BBB [81]. However, the subsequent restoration of BBB integrity and the presence of elevated intracranial pressure impose significant constraints on the timing, size, and distribution of nanomaterials delivered to the affected brain regions [44]. Although transient BBB permeability facilitates size-selective accumulation of nanomaterials within the damaged tissue—an effect analogous to the “enhanced permeation and retention” (EPR) phenomenon—the therapeutic window for effective nanomaterial delivery remains narrow, limited to a few hours post-injury. Unlike chronic conditions such as cancer or arthritis, which feature sustained BBB disruption and thus allow a more extended window for nanomaterial-based intervention [43], TBIs present an acute scenario requiring precise temporal coordination. In this context, nanozyme delivery must be tightly regulated and can occur through three distinct mechanisms. First, the cross-linked antioxidant nanozyme (cl-nanozyme) achieves delivery via thrombus incorporation. Second, the neutrophil-like cell-membrane-coated mesoporous Prussian blue nanozyme (MPBzyme@NCM) traverses the BBB through receptor-mediated transcytosis, aided by adhesion molecules such as intercellular adhesion molecule-1 (ICAM-1). Finally, both PNzyme/MnO₂ and EMT-nanozyme penetrate the BBB using transferrin (Tf)-mediated endocytosis (Fig. 4b).

Over the past several decades, animal models have been fundamental to elucidating the pathophysiology of TBI and to evaluating potential therapeutic interventions. A variety of experimental paradigms have been established to model key features of human TBI—including focal, diffuse, penetrating, and blast injuries—across species ranging from rodents to large animals. Among these, the controlled cortical impact (CCI) model is widely employed for its versatility and reproducibility. Larger animal models address questions of developmental stage and translational relevance, while closed-head and blast injury models, such as CHIMERA and WRAIR, are valuable for studying concussion and mild TBI mechanisms [83–93]. Recent advances, including the use of blood-based biomarkers to better align animal models with human TBI, may help accelerate clinical translation. However, these approaches still require rigorous validation, and there remains an urgent need for innovative therapies to overcome current barriers in TBI treatment. Despite promising neuroprotective effects observed in animal models, multiple phase II and III clinical trials have failed to demonstrate significant benefit in patients with TBI [94–96]. Notably, randomized controlled trials of progesterone (ProTECT, ProTECT III, SYNAPSE) failed to improve neurological outcomes or survival, and similar results were seen in trials of moderate hypothermia in children—including both phase II and phase III studies—as well as in studies of corticosteroids (MRC CRASH trial), all of which did not show clinical benefit [97–104]. These repeated failures highlight the considerable challenges in translating preclinical success into clinical efficacy for TBI, and emphasize the urgent need for more predictive models and innovative therapeutic strategies to improve outcomes in this population.

### Nano-driven approaches for TBI treatment

**Fig. 5:**
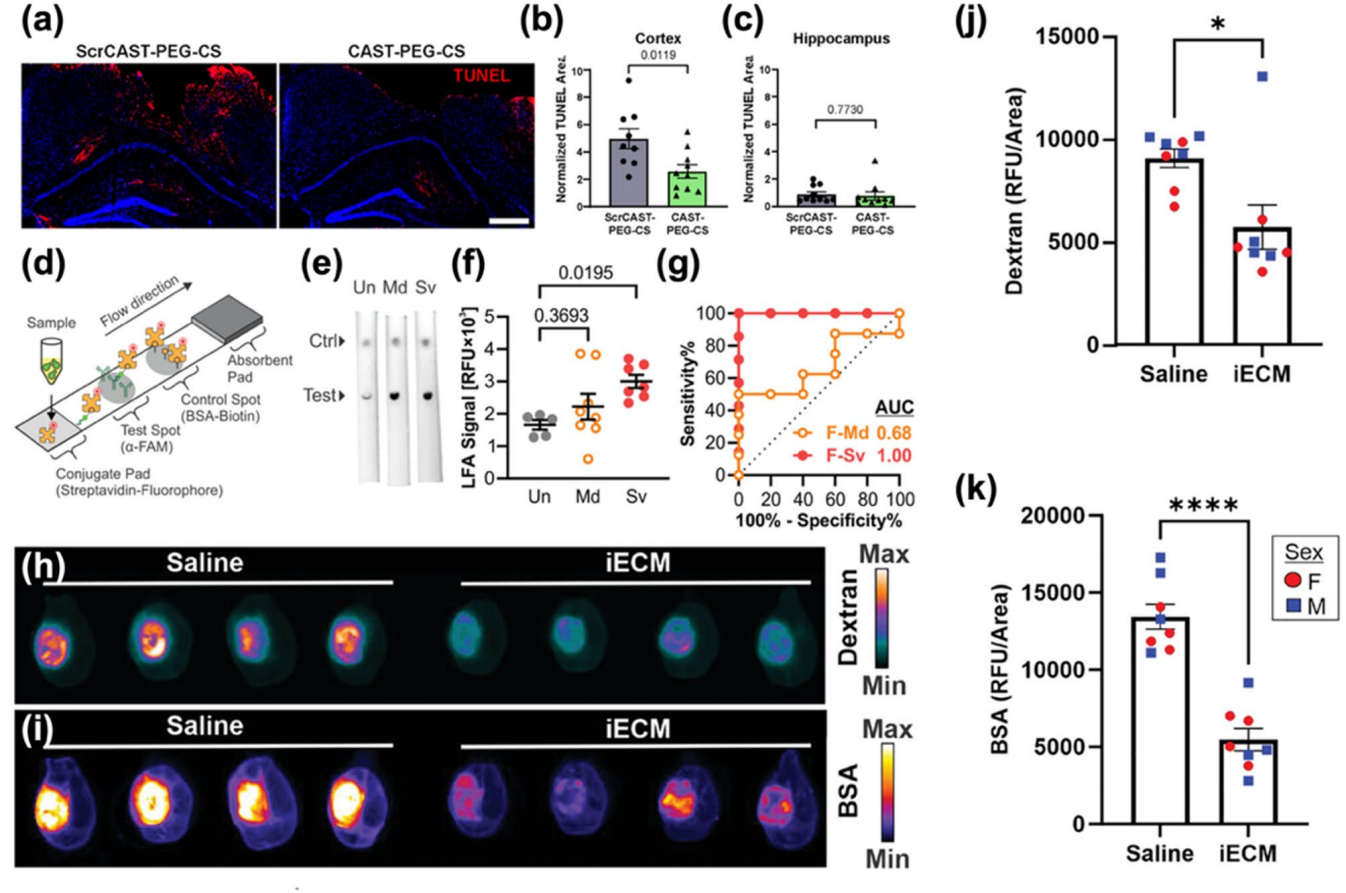
Diverse nanomaterial-based TBI treatment models. (**a**) TUNEL staining of coronal brain sections from mice treated with ScrCAST–PEG–CS or CAST–PEG–CS (red: TUNEL; blue: nuclei; scale bar = 500 μm). (**b**,** c**) Quantification of TUNEL-positive areas normalized by nuclear area in the injured cortex and hippocampus following treatment (*n* = 10 mice; mean ± SEM; unpaired t-test). Copyright (2024) American Chemical Society. (**d**) Schematic illustration of the lateral flow assay (LFA) designed to measure c-Peptide in samples. (**e**) Representative fluorescence images of LFAs for urine samples (Ctrl = control). **(f)** Quantification of urinary c-peptide levels at 1 h post-injection, determined by the integrated signal of the test spot. (**g**) Corresponding ROC curves for the assay. Data are presented for female mice (*n* = 5 for F-Un, *n* = 7 for F-Sv, *n* = 8 for F-Md; mean ± SE, analyzed by one-way ANOVA with Dunnett’s post-hoc test versus uninjured control). Copyright (2023) Wiley. **(h**) Representative LiCor Odyssey scan of 10 kDa dextran following treatment with iECM or saline control. (**i**) Representative LiCor Odyssey scan of BSA following treatment with iECM or saline control. (**j**) iECM reducing the permeability of 10 kDa dextran into the injured brain region in both male (blue square) and female (red circle) mice (*n* = 8; mean ± SEM; unpaired two-tailed t-test, *p < 0.05). (**k**) iECM reducing the permeability of BSA into the injured brain region in both male (blue square) and female (red circle) mice (*n* = 8; mean ± SEM; unpaired two-tailed t-test, ****p < 0.0001). Copyright (2023) Wiley

TBIs present formidable therapeutic challenges, attributable to their inherent heterogeneity and the complex cascade of pathogenic processes initiated by primary injury. Primary lesions—including focal intracranial hemorrhage, epidural and subdural hematomas, cerebral contusions, and direct axonal disruption—contribute to substantial variability in molecular and cellular responses, thereby complicating the precise targeting of secondary injury mechanisms. Moreover, the multifactorial pathology that unfolds in the aftermath of brain injury significantly constrains the efficacy of existing diagnostic and therapeutic approaches. Notably, access to the injured brain is further restricted by the BBB, which poses a major obstacle to the delivery of both small-molecule and macromolecular therapeutics [105–107]. In this context, nanomaterial-based platforms have emerged as highly promising agents, offering innovative strategies that address the limitations of conventional therapies. Among these, nanotheranostics have demonstrated efficacy in targeting calpain activity within critical brain regions, such as the cortex and hippocampus, in the aftermath of TBIs. Notably, although calpain activity was reduced in both regions, the inhibition of apoptosis was significantly more pronounced in cortical neurons compared to those in the hippocampus, suggesting regional variability in therapeutic response to calpain inhibition [69, 108, 109]. After 1 h of administration of either calpastatin peptide (CAST) –polyethylene glycol (PEG)–calpain sensor (CS) or scrambled CAST peptide–PEG–CS, the brain sections were analyzed using terminal deoxynucleotidyl transferase biotin dUTP nick end labeling (TUNEL) staining to assess apoptosis (Fig. 5a). Quantitative analysis of TUNEL-positive areas demonstrated that CAST treatment reduced the number of apoptotic cells in the injured cortex by approximately 50% (Fig. 5b), whereas no significant reduction was observed in the hippocampus (Fig. 5c). In parallel, Kudryashev and co-workers developed a minimally invasive nanosensor capable of detecting calpain activity through blood and urine biomarkers [109]. This nanosensor exhibited enhanced sensitivity compared to conventional biomarkers, such as glial fibrillary acidic protein (GFAP), especially in identifying mild TBIs. Additionally, a lateral flow assay (LFA) was designed as an alternative immunoassay format, enabling rapid biomarker detection (20 min versus 3 h for enzyme-linked immunosorbent assay (ELISA), albeit with a reduction in sensitivity (Fig. 5d). The LFA employs a sandwich format in which an α-FAM antibody is immobilized on a nitrocellulose membrane to capture FAM-tagged c-Peptide, while fluorescently labeled streptavidin on a conjugate pad detects the biotin moiety, with bovine serum albumin (BSA)-biotin serving as a positive control. When applied in a dipstick format to diluted urine samples, the assay detected peptide concentrations ranging from 3.9 to 250 nM (Fig. 5e). Notably, urine samples from female mice with severe injury produced substantially elevated LFA signals, achieving a receiver operating characteristic (ROC) area under the curve (AUC) of 1.00. In contrast, samples from female mice with mild injury and from male mice at either injury severity exhibited diminished diagnostic performance (ROC AUCs: 0.68). (Figure 5f and g). Collectively, these findings highlight the potential of this non-invasive approach for the early diagnosis and monitoring of TBI progression. These advancements also emphasize ongoing efforts to refine calpain inhibition as a therapeutic strategy, addressing current limitations and aiming to improve outcomes in TBI management. The integration of targeted inhibition with advanced diagnostic tools represents a significant step forward in optimizing neuroprotective interventions for TBIs. Furthermore, nanozymes with selectivity for ROS and reactive nitrogen species (RNS) have been employed to scavenge these harmful species, thereby reducing inflammation levels in brain lesions [110–113]. Recent advancements have also focused on exploiting disruptions in the BBB to enhance drug delivery. Diaz et al. [108] introduced an infusible extracellular matrix-derived biomaterial (iECM) that promotes vascular integrity and modulates the inflammatory response in TBIs. The iECM localized at the injury site and reduced vascular permeability in a dose-dependent manner, improving BBB function and decreasing the extravasation of molecules into the brain. Mice were perfused, and organs were harvested 20 min after tracer injection. Whole-brain imaging was then performed to assess the extent of tracer extravasation into the injured region. Analysis of the tracer signal in the injured cortex revealed that, following iECM delivery, the extravasation of BSA and dextran was significantly reduced by approximately 2.5-fold and 1.6-fold, respectively, compared to saline controls. This reduction in tracer leakage was consistent across both iECM-treated male and female mice, with no sex-based differences observed. These findings suggest that iECM preserves vascular integrity in the brain across a range of molecular sizes (at least 10–66 kDa) (Fig. 5h and k). Moreover, the administration of iECM modulated gene expression, specifically in pathways associated with neuroinflammation and neuroprotection, underscoring its potential as a therapeutic tool for ameliorating BBB dysfunction in TBI. iECM not only enhances drug delivery across the disrupted BBB but also promotes vascular integrity and modulates inflammatory response, providing a comprehensive approach to TBI treatment [114, 115]. Collectively, these nano-based materials and strategies emphasize the remarkable potential of nanotechnology in advancing TBI therapy. By addressing critical challenges such as targeted drug delivery, inflammation reduction, and early diagnostic capabilities, nano-based approaches offer a multifaceted and effective means of improving clinical outcomes for individuals suffering from TBIs.

In summary, TBI is a multifaceted condition resulting from various etiologies, such as falls, vehicular accidents, sports injuries, and military blast exposures, which induce both immediate mechanical damage and delayed secondary injury processes. These secondary mechanisms, including oxidative stress, excitotoxicity, neuroinflammation, and BBB disruption, further exacerbate neuronal loss and functional decline. In response to these challenges, nanomaterials have emerged as a promising therapeutic avenue. Their unique properties enable targeted drug delivery, effective scavenging of reactive species, and precise modulation of inflammatory pathways. Moreover, innovations such as multifunctional nanozymes and diagnostic nanosensors present new opportunities for early intervention and enhanced treatment efficacy. Collectively, these advancements highlight the potential of nanotechnology to address the limitations of conventional therapies and ultimately improve outcomes for individuals with TBIs.

**Table 1:** Overview of nanotherapeutic approaches for TBI with key features and therapeutic strategies

Chapter	Materials	Key features & therapeutic strategy	Applications
3.1	PEGylated-polystyrene nanoparticles	- Passive targeting via BBB disruption	Acute-phase drug delivery leveraging transient BBB opening
		- PEGylation to prolong circulation time	
		- Size-dependent accumulation at the TBI site	
3.2	pSi nanoparticles	- ECM-targeted delivery via CAQK peptide	Targeted delivery to the injury microenvironment
		- Binds to CSPGs overexpressed in the injured brain	
3.3	Carbon dot nanoparticles	- Carbogenic nanozyme function	Long-term oxidative stress regulation and neuroprotection
		- Scavenges ROS and RNS	
		- Reduces oxidative stress and neuroinflammation	
3.4	Dendrimer nanoparticles	- PAMAM dendrimer-based platform	Suppression of early neuroinflammatory response to prevent secondary injury
		- Sinomenine conjugation for anti-inflammation	
		- Targets microglia and suppresses neuroinflammation	
3.5	Lipid nanoparticles	- CAQK peptide-functionalized, ROS-responsive LNP: ROS scavenging & Ca²⁺ overload inhibition	Acute-phase therapeutic delivery and prolonged circulation for sustained drug exposure
		- PEGylation density-optimized LNP: prolonged circulation, enhanced brain accumulation	
3.6	siRNA-based nanoparticles	- PEGylated multimeric RNA nanoparticles	Gene silencing therapy to inhibit pro-inflammatory cytokine expression
		- RNAi-mediated TNF-α silencing	
		- Rolling circle transcription-based fabrication	
		- Enhanced BBB penetration and gene knockdown	

BBB: blood-brain-barrier ECM: Extracellular matrix. CSPGs: Chondroitin sulfate proteoglycan. ROS: Reactive oxygen species. RNS: Reactive nitrogen species. PAMAM: Poly(amidoamine). LNP: Lipid nanoparticle

## Nanotherapeutics for TBI

In this chapter, we summarize the nanomaterials-based therapeutic approaches aimed at overcoming TBIs. As a concrete plan for the above strategy, the chapter was organized by considering the size of the particles after primary damage, the timing of administration, drug delivery and accumulation, BBB passage, and clinical aspects of genetically based nanoparticles from the perspective of therapeutics. The chapter consists of six sections: (1) PEGylated-polystyrene nanoparticles, (2) Porous silicon nanoparticles, (3) Lysine-based nanoparticles, (4) Dendrimer nanoparticles, (5) LNPs, and (6) siRNA-based nanoparticles (Table 1).

### PEGylated-polystyrene nanoparticles

**Fig. 6:**
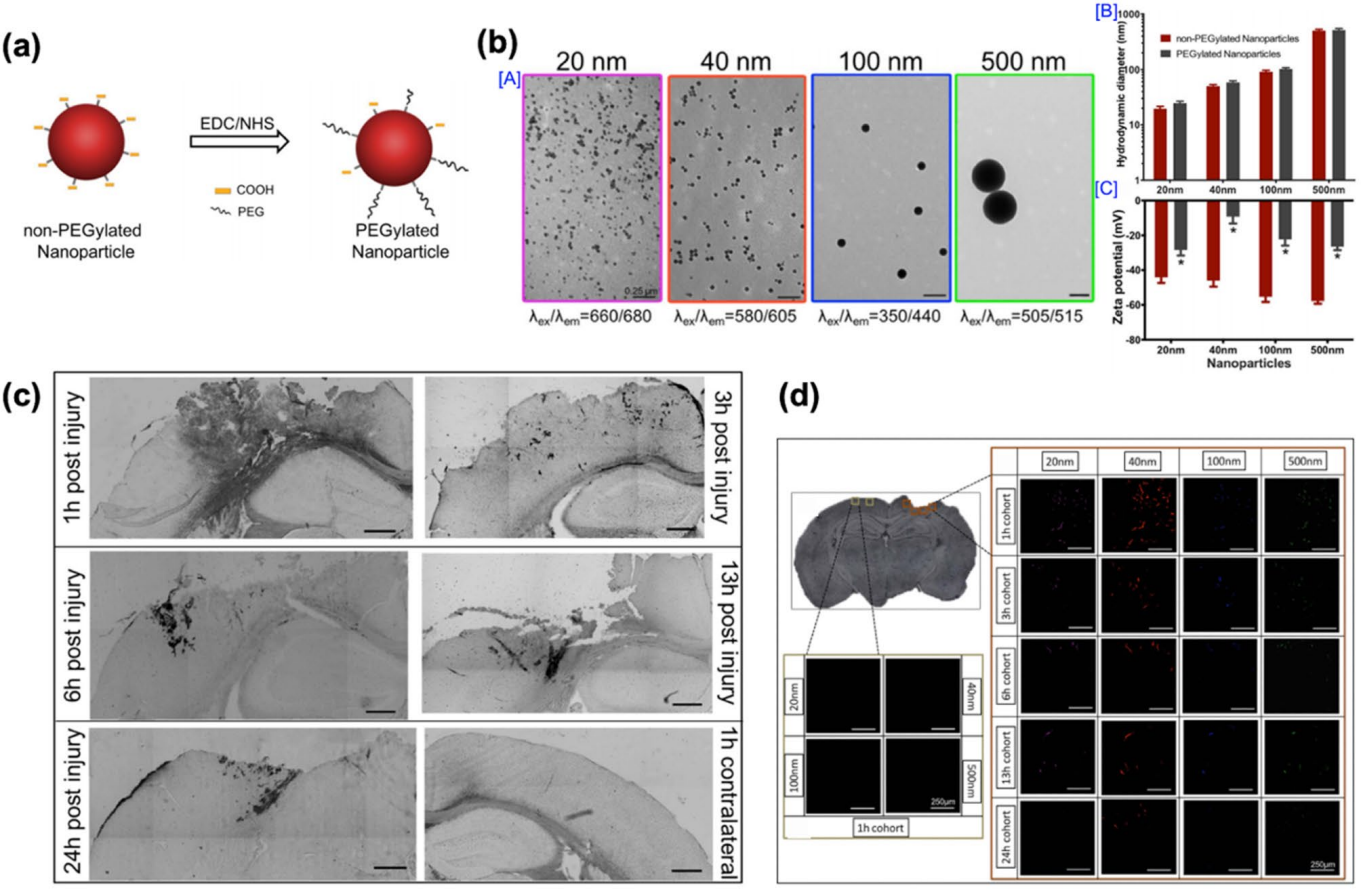
PEGylated nanoparticles for therapeutic strategies in TBI treatment. (**a**) Schematic representation of PEG conjugation to nanoparticles via EDC/NHS chemistry. **(b**) [A] TEM images of monodisperse PEGylated nanoparticles with sizes ranging from 20 to 500 nm. [B] Hydrodynamic diameters of both non-PEGylated and PEGylated nanoparticles. [C] Zeta potential measurements for both non-PEGylated and PEGylated nanoparticle groups (*n* = 3, mean ± SEM, *p < 0.05, student’s t-test). (**c**) Representative images of the extravasation of HRP in the injured region at 1–24 h post-injury. (**d**) Temporal accumulation of nanoparticles of varying sizes in the injured brain tissue following a TBI. Scale bar = 250 μm [78]. Copyright (2015) Springer Nature

The first example is a PEGylated polystyrene nanoparticle (PSNP) formulation, reported by Bharadwaj and co-workers in 2015 (Fig. 6). This study aimed to investigate the effects of nanoparticle size and administration timing on the passive accumulation of nanoparticles in the injured brain following TBI. The researchers hypothesized that PEGylation would improve the in vivo circulation time of nanoparticles, thereby facilitating their accumulation at the injury site during the transient permeability of the BBB.

The PSNPs were synthesized by conjugating methoxy-polyethylene glycol-amine (mPEG-NH₂) to carboxylated polystyrene nanoparticles through EDC/NHS coupling chemistry (Fig. 6a). The PEG derivative, mPEG, which has a methoxy group at one end, is widely utilized in nanoparticle design to improve colloidal stability, extend systemic circulation time, and minimize immune recognition. In this study, mPEG was employed to introduce PEG chains on the surface of PSNP, thereby enhancing their pharmacokinetic profile. Carboxylated polystyrene was selected as the core material due to its well-defined surface chemistry and ease of PEGylation. Four different nanoparticle sizes (20, 40, 100, and 500 nm) were prepared, and the resulting PSNPs exhibited an increased hydrodynamic diameter and a reduced zeta potential compared to the non-PEGylated nanoparticles (Fig. 6b). Transmission electron microscopy (TEM) images confirmed their spherical morphology and distinct fluorescence emission profiles were obtained for each particle size, allowing multicolor imaging. To determine the optimal time window for nanoparticle delivery, in vivo biodistribution studies were conducted using a CCI mouse model, a widely used TBI model that induces focal cortical injury with reproducible primary and secondary injury features. PSNPs were intravenously administered at various time points (1–24 h post-injury). Fluorescence imaging and quantitative analysis revealed that nanoparticle accumulation in the injured brain peaked when administered 1 h post-injury and remained significant up to 6 h (Fig. 6c). Notably, smaller nanoparticles (20 and 40 nm) exhibited higher and more prolonged retention at the injury site compared to larger nanoparticles (100 and 500 nm) (Fig. 6d).

This study offers compelling evidence that passive nanoparticle accumulation following TBI is influenced by both particle size and the timing of administration. Nanoparticles smaller than 100 nm demonstrated enhanced accumulation and prolonged retention within the injured brain. These findings provide valuable insights for designing nanotherapeutic systems that leverage the transient disruption of BBB immediately after injury, thereby optimizing drug delivery efficiency and improving therapeutic outcomes.

### Porous silicon nanoparticles

**Fig. 7:**
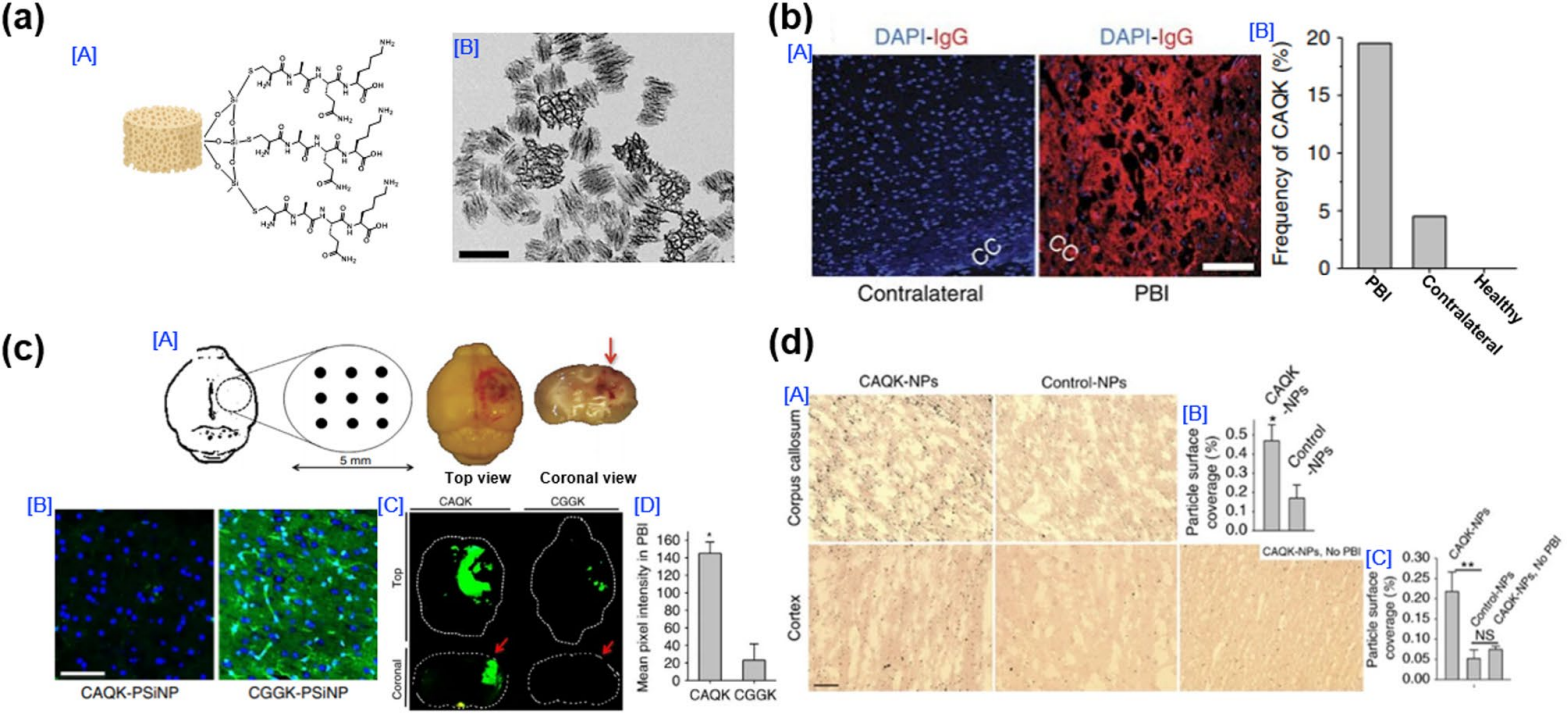
Targeting-peptide-decorated nanoparticles for TBI treatment. (**a**) [A] Structure of pSiNPs and [B] TEM image of peptide-conjugated pSiNPs. (**b**) [A] Immunofluorescence images demonstrating leakage through the compromised BBB in penetrating brain injury (PBI), with perfused PBI brain tissue stained in the region around the corpus callosum for mouse IgG (red) and 4,6-diamidino-2-phenylindole (blue: DAPI; Scale bar = 50 mm). [B] CAQK phage frequency in the brain as a percent of total phage recovered. (**c**) [A] Perfused brain images, 6 h after unilateral injury, showing top and internal coronal view. [B] Magnified fluorescence images of GFP expression in PBI brain tissue. [C] Fluorescence brain images (top and coronal views) and mean pixel intensity in PBI of mice injected with FAM-labelled peptides 6 h post-PBI (*n* = 6; mean ± SEM; two-tailed heteroscedastic Student’s test; *p < 0.05). (**d**) [A-C] CAQK-conjugated AgNPs (CAQK-NPs) exhibiting enhanced binding to sections from the corpus callosum and cortex of human brains with traumatic brain injury (TBI), compared to normal brain tissue or control nanoparticles. Scale bar = 50 μm [114]. Copyright (2016) Springer Nature

The second example involves a porous silicon nanoparticle (pSiNP) formulation functionalized with the CAQK peptide, reported by Cheng and co-workers in 2016 (Fig. 7). This study introduced an extracellular matrix (ECM)-targeted delivery platform for TBI, with a specific focus on penetrating brain injury (PBI), a type of TBI characterized by focal mechanical damage and glial scarring. The design of the material was based on the observation that ECM components, such as tenascin-C, versican, and chondroitin sulfate proteoglycans (CSPGs), are prominently exposed following a TBI. In response to primary injury, the brain undergoes a repair process involving glial scar formation, during which CSPGs are substantially upregulated as part of ECM remodeling. While this process helps to limit secondary injury by stabilizing the lesion site and preventing further tissue damage, the accumulation of CSPGs also constitutes a physical and biochemical barrier to axonal regeneration, thus impeding long-term neurorepair. As key components of the glial scar, CSPGs are considered pathological hallmarks of the injured brain microenvironment. The CAQK peptide identified through in vivo phage display selectively binds to these injury-associated ECM structures. The authors hypothesized that the conjugation of CAQK to PSNPs would facilitate systemic, injury-targeted delivery, overcoming the inhibitory microenvironment and enhancing the therapeutic accumulation at the injury site. The pSiNPs were synthesized by conjugating PEG-NH₂ and the CAQK peptide to carboxylated pSiNPs via EDC/NHS coupling chemistry (Fig. 7a [A]). TEM analysis confirmed the spherical morphology and monodispersed nature of the pSiNPs (Fig. 7a [B]). The CAQK peptide was successfully conjugated to the surface of the pSiNPs without compromising the particle’s stability. The specificity of the CAQK peptide binding was further validated by analyzing brain tissue from mice after a TBI. Immunohistochemical staining revealed a significantly higher presence of CAQK-binding ECM in the injured hemisphere compared to the contralateral side and healthy control brains (Fig. 7b [A], [B]). In vivo evaluation was performed using a CCI mouse model. Following intravenous administration, the accumulation of CAQK-pSiNPs in the injured cortex was significantly higher than that of control pSiNPs functionalized with a scrambled CGGK peptide (Fig. 7c [B]). Fluorescence imaging and quantitative analysis further confirmed the preferential localization of CAQK-pSiNPs at the injury site, most notably in the cortex and corpus callosum (Fig. 7c [C], [D]). Importantly, the translational relevance of this ECM-targeted strategy was supported through analysis of human TBI brain tissue. Histological examination demonstrated a prominent accumulation of CAQK-pSiNPs in the corpus callosum and cortex of human samples obtained from TBI patients, whereas non-targeted pSiNPs exhibited negligible localization (Fig. 7d [A]). Quantitative measurement confirmed a significantly higher particle coverage in the injured areas of human brain tissue when CAQK peptides were employed (Fig. 7d [B], [C]). This study provides compelling evidence that ECM-targeted delivery using CAQK peptide-functionalized pSiNps enables systemic, non-invasive nanoparticle accumulation at the TBI site. Furthermore, the successful application of this strategy in human brain tissue samples highlights its potential translational value. These findings suggest that CAQK-functionalized pSiNPs can serve as a versatile platform for the targeted delivery of therapeutic and diagnostic agents in TBI treatment and may offer promising opportunities for future clinical development aimed at improving precision medicine strategies for brain injury.

### Carbon Dot nanoparticles

**Fig. 8:**
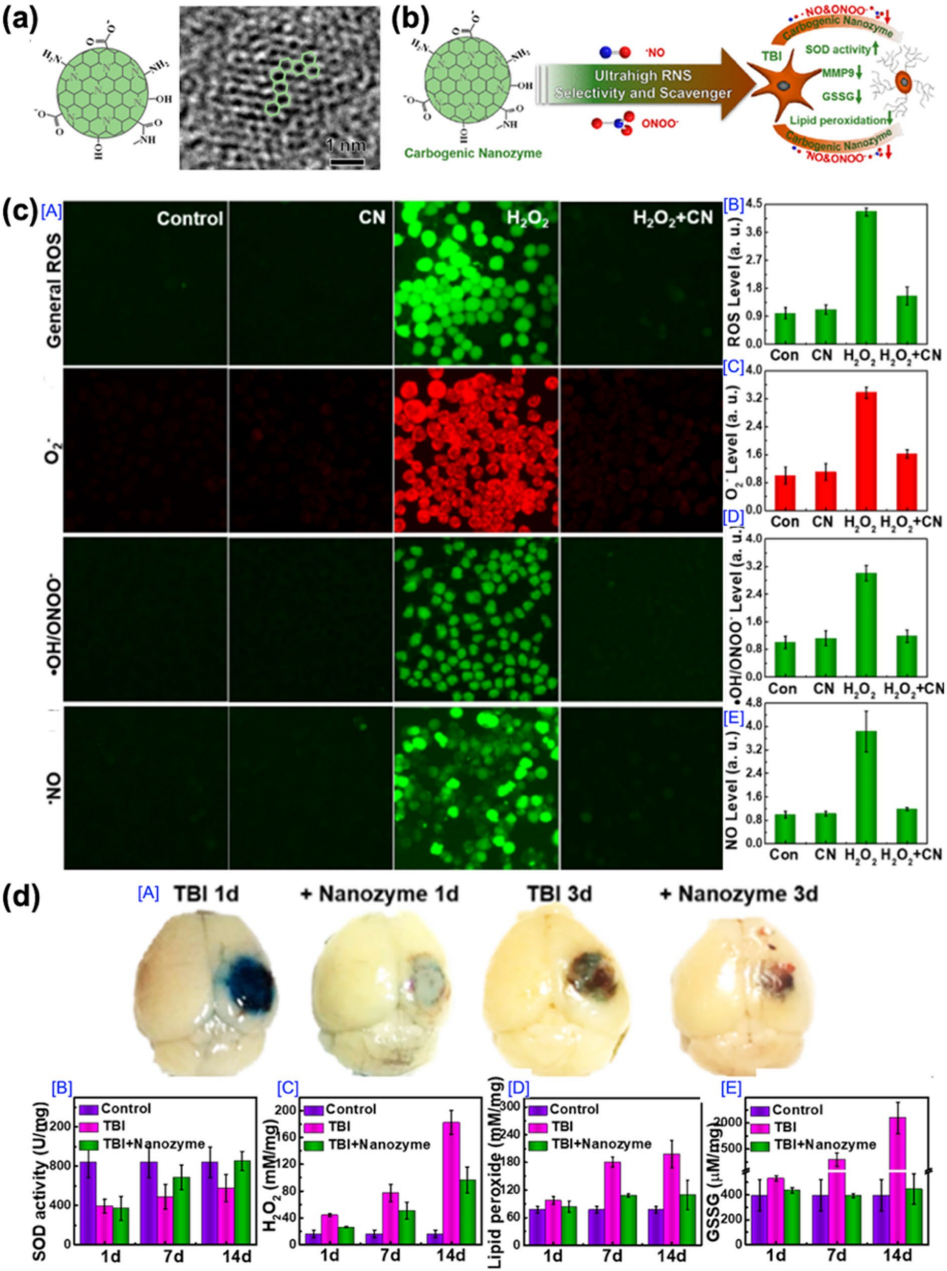
Application of carbon dot nanozyme in TBI Treatment. (**a**) Illustration of the structure of the carbogenic nanozyme and its TEM image. (**b**) Action mechanism of carbogenic nanozyme related with RNS, intermediate products that contribute to ongoing damage in the nervous system during the progression of traumatic brain injury (TBI). (**c**) [A] In vitro assessment of RONS scavenging activity by the carbogenic nanozyme and its protective effects against H_2_O_2_-induced apoptosis in N2a cells. Quantitative flow cytometry analyses of intracellular levels of [B] general ROS, [C] O_2_^•−^, [D] •OH/ONOO^−^, and [E] •NO under various treatment conditions. (**d**) [A] Optical images of brains from TBI mice treated with or without the carbogenic nanozyme. Quantitative analyses of oxidative stress-related indicators, including [B] SOD, [C] H_2_O_2_, [D] lipid peroxidation, and [E] GSSG concentrations, in TBI mice with or without nanozyme treatment [116]. Copyright (2019) American Chemical Society

The third example is a carbon dot nanoparticle system reported by Xiao-Dong Zhang and co-workers in 2019 (Fig. 8). This study introduced a novel nanozyme strategy that integrates nanomaterials with catalytic activity to selectively regulate oxidative stress in TBIs. The design of these carbon dot-based nanoparticles was grounded in the concept of mimicking enzymatic activity with nanomaterials. Nanozymes are nanoscale materials that possess enzyme-like catalytic properties, offering several advantages over conventional enzymes, including enhanced stability under physiological conditions, resistance to environmental variations (such as pH and temperature), cost-effectiveness, and scalability in production. Given the central role of oxidative stress in the secondary injury mechanism of TBIs, the authors sought to develop carbon dot nanoparticles with tailored catalytic activity capable of selectively eliminating RNS, while preserving the physiological ROS signaling. Furthermore, these nanoparticles were designed to simultaneously scavenge both ROS and RNS, collectively referred to as reactive oxygen and nitrogen species (RONS), which are responsible for sustained neuroinflammation and neuronal damage following TBI. The carbon dot nanoparticles were synthesized through a hydrothermal carbonization process, using citric acid and cysteine as precursors (Fig. 8a [A]). This approach yielded the synthesis of monodisperse, spherical carbon dot nanoparticles with an average hydrodynamic diameter of approximately 3 nm, as confirmed by high-resolution TEM imaging (Fig. 8a [B]). The surface of these carbon dot nanoparticles was enriched with amino and thiol functional groups, which contributed to their catalytic activity and selective targeting of RNS. The therapeutic mechanism of these nanoparticles, as illustrated schematically, involves their ability to selectively scavenge RNS species, thereby mitigating oxidative damage. By reducing lipid peroxidation, inhibiting matrix metalloproteinase (MMP) activity, and enhancing superoxide dismutase (SOD) activity, the carbon dot nanoparticles effectively attenuate secondary injury following a TBI (Fig. 8b).

The physicochemical and catalytic properties of the carbon dot nanoparticles were systematically characterized. In vitro assays demonstrated that the carbon dot nanoparticles exhibited robust scavenging activity against RNS, particularly nitric oxide (•NO) and peroxynitrite (ONOO^−)^, while displaying minimal reactivity toward general ROS, such as superoxide anion (O_2_^−^) and hydrogen peroxide (H_2_O_2_) (Fig. 8c [A]). Quantitative analyses confirmed that treatment with the carbon dot nanoparticles significantly reduced intracellular levels of general ROS (Fig. 8c [B]), O_2_^−^ (Fig. 8c [C]), and ONOO^−^ (Fig. 8c [D]), compared to control and H_2_O_2_-treated groups. Moreover, the nanoparticles effectively suppressed •NO levels under oxidative stress conditions (Fig. 8c [E]). The in vivo therapeutic efficacy was evaluated using a CCI mouse model. Following intravenous administration, the carbon dot nanoparticles preferentially accumulated at the injury site due to the transient disruption of the BBB after a TBI. Macroscopic examination of brain tissue demonstrated a marked reduction in lesion size at both 1 and 3 days post-injury in treated animals compared to untreated TBI controls (Fig. 8d [A]). Biochemical analysis revealed that treatment restored superoxide dismutase (SOD) activity (Fig. 8d [B]), while decreasing H_2_O_2_ concentration (Fig. 8d [C]), lipid peroxidation (Fig. 8d [D]), and glutathione disulfide (GSSG) levels (Fig. 8d [E]) over a 14-day period.

This study provides compelling evidence that carbon dot nanoparticles with ultrahigh selectivity for RNS can effectively regulate oxidative stress and attenuate secondary brain injury following TBI. Their small particle size, excellent biocompatibility, and precise RNS targeting underscore the potential of this platform as a promising nanotherapeutic approach. Moreover, the successful application of enzyme-mimicking nanomaterials in neurotrauma highlights the versatility of nanozyme-based strategies and suggests that their continued development may offer valuable clinical solutions for TBI treatment.

### Dendrimer nanoparticles

**Fig. 9:**
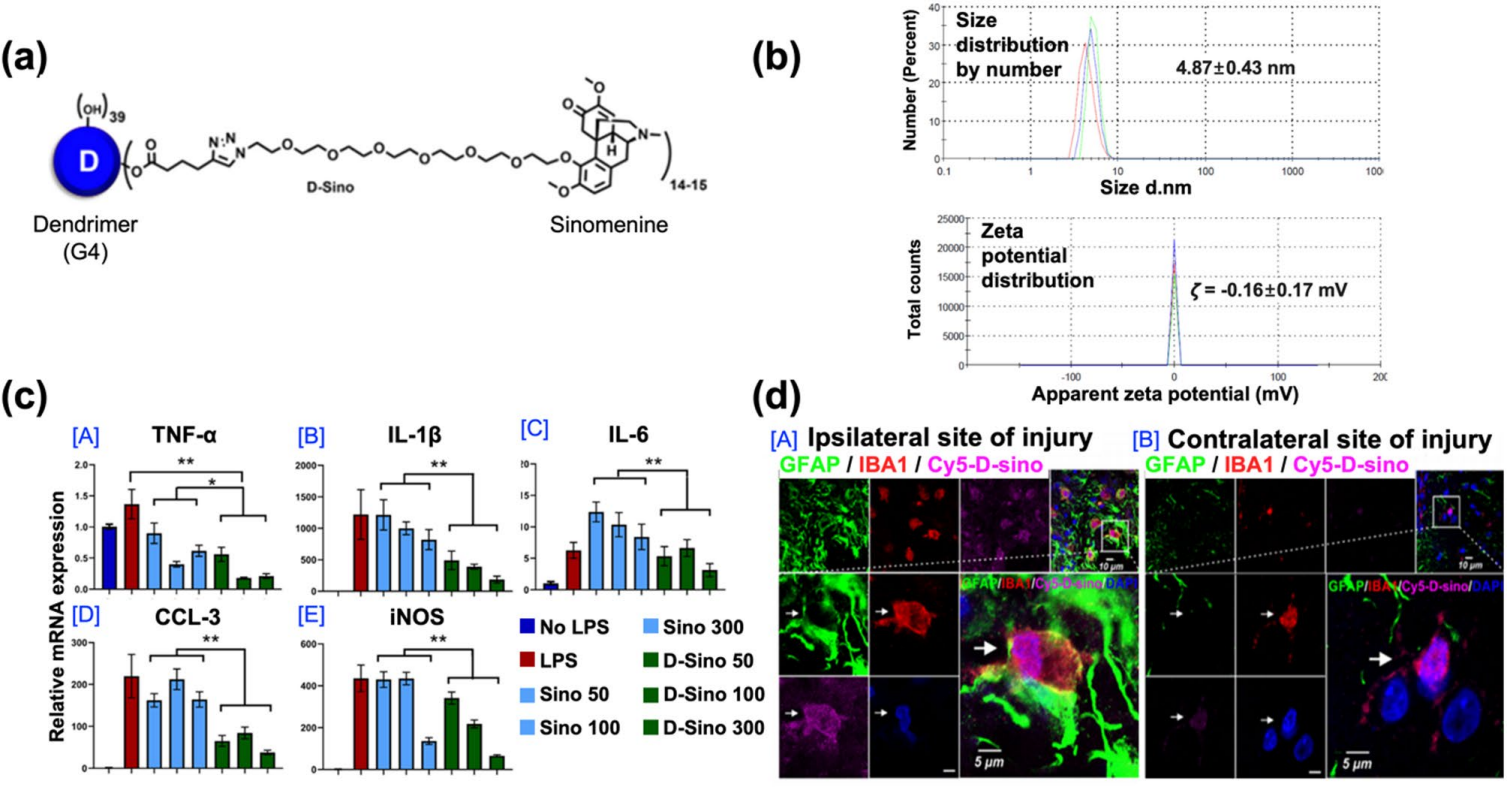
Dendrimer-based nanoparticle system for TBI treatment. (**a**) The structure of the nanoformulation. (**b**) Size distribution and zeta potential measurements of the nanoformulation. (**c**) Cytokine expression levels using RT-qPCR in cells pretreated with D-Sino or free Sino, followed by LPS activation (early/acute inflammation) (mean ± SEM; *p < 0 0.05, **p < 0 0.01; one-way ANOVA with Bonferroni post hoc test). TNF-α: Tumor necrosis factor-α; IL-1β: Interleukin-1β; IL-6: Interleukin-6; CCL3: Chemokine ligand 3; iNOS: inducible nitric oxide synthase. (**d**) In vivo cellular localization of Cy5-D-Sino. Brain tissue slices were stained to visualize Cy5-D-Sino (magenta), astrocyte (green; GFAP), microglia (red; IBA1), and nuclei (blue; DAPI). (A) Co-localization of Cy5-D-Sino, astrocytes (GFAP + cells) and microglia (IBA1 + cells) at the injured ipsilateral site of the CCI. (B) Co-localization of Cy5-D-Sino, astrocytes (GFAP + cells), and microglia (IBA1 + cells) in the contralateral cortex of the CCI. Boxed regions indicate areas of interest shown at higher magnification. Arrows indicate co-localized Cy5-D-Sino, astrocyte, and microglia in both the ipsilateral and contralateral sites of injury (scale bars = 10 μm and 5 μm) [112]. Copyright (2020) Elsevier

The fourth example is a dendrimer-based nanoparticle system developed by Xiao-Bing Fu and co-workers in 2020 (Fig. 9). This study introduced a microglia-targeted nanotherapeutic strategy for the treatment of acute neuroinflammation in TBI using dendrimer nanoparticles conjugated with sinomenine (SIN), an anti-inflammatory alkaloid. The design was based on the critical role of activated microglia in driving the secondary injury cascade following a TBI. Upon activation, the microglia releases pro-inflammatory cytokines and mediators that exacerbate neuronal damage. To address this, the authors aimed to develop dendrimer nanoparticles capable of selectively targeting activated microglia and delivering SIN to suppress neuroinflammatory responses, while minimizing systemic side effects. Dendrimers are nanoscale, highly branched, and symmetrical macromolecules that originate from a central core and expand through multiple layers of branching units. Their well-defined architecture and multivalency make them ideal platforms for drug delivery, offering high payload capacity, controlled drug release, and surface functionalization for targeted delivery. Leveraging these properties, the authors designed dendrimer nanoparticles to deliver SIN specifically to activated microglia in the injured brain.

The dendrimer nanoparticles were synthesized using a generation 4 (G4) poly(amidoamine) (PAMAM) dendrimer as the core structure (Fig. 9a [A]). SIN was conjugated to the dendrimer via a copper (Cu)-catalyzed click reaction using a PEG linker, which was further modified with an acid-sensitive hydrazone bond. This dual-modification strategy enabled efficient drug conjugation and pH-responsive drug release in the acidic microenvironment characteristic of activated microglia. Successful conjugation of SIN to the dendrimer backbone was confirmed through structural characterization. DLS analysis revealed a uniform hydrodynamic diameter of approximately 4.87 nm and a zeta-potential of − 0.16 mV (Fig. 9b). The anti-inflammatory efficacy of the SIN-conjugated dendrimer nanoparticles was assessed in vitro using lipopolysaccharide (LPS)-stimulated microglial cells. Treatment with the dendrimer nanoparticles significantly downregulated the mRNA expression of pro-inflammatory cytokines, including TNF-α, IL-1β, IL-6, CCL-3, and iNOS, compared to free SIN or control groups (Fig. 9c [A]–[E]). These results confirmed the ability of the dendrimer nanoparticles to effectively attenuate microglial activation.

In vivo, therapeutic evaluation was conducted using a CCI mouse model. Following intravenous administration, fluorescence imaging demonstrated that the dendrimer nanoparticles preferentially accumulated at the ipsilateral injury site, where they co-localized with activated microglia (IBA1-positive cells) and astrocytes (GFAP-positive cells). In contrast, minimal accumulation was observed in the contralateral brain region (Fig. 9d [A], [B]). These findings indicated the effective targeting capability of the dendrimer nanoparticles toward inflamed brain tissue. This study provides compelling evidence that microglia-targeted delivery of anti-inflammatory agents via dendrimer nanoparticles can effectively suppress neuroinflammation and attenuate secondary brain injury following TBI. The acid-sensitive drug release mechanism, selective microglial uptake, and favorable pharmacokinetic profiles highlight the potential of this nanotherapeutic platform. These findings support that dendrimer nanoparticles can contribute to the development of clinically translatable strategies for precision therapy targeting neuroinflammation in TBIs.

### Lipid nanoparticles

The fifth example, reported in 2022 by Hongmei Liu and co-workers, introduced LNP-based strategy to regulate oxidative stress and calcium overload in TBIs. The sixth example, reported in 2023 by Ester J. Kwon and co-workers, presented an LNP platform designed to optimize pharmacokinetics and enhance targeted delivery to the injured regions of the brain by modulating PEGylation density. These two studies provide distinct yet complementary approaches that leverage the versatility of LNPs to address multiple aspects of TBI pathology. LNPs have emerged as a promising nanoplatform for TBI treatment due to their biocompatibility, structural flexibility, and ability to exploit pathological features of TBIs, such as BBB disruption and oxidative stress, for targeted therapeutic delivery. In particular, LNPs can be engineered with functional moieties and responsive elements to mitigate secondary injury processes, including oxidative stress and neuroinflammation.

**Fig. 10:**
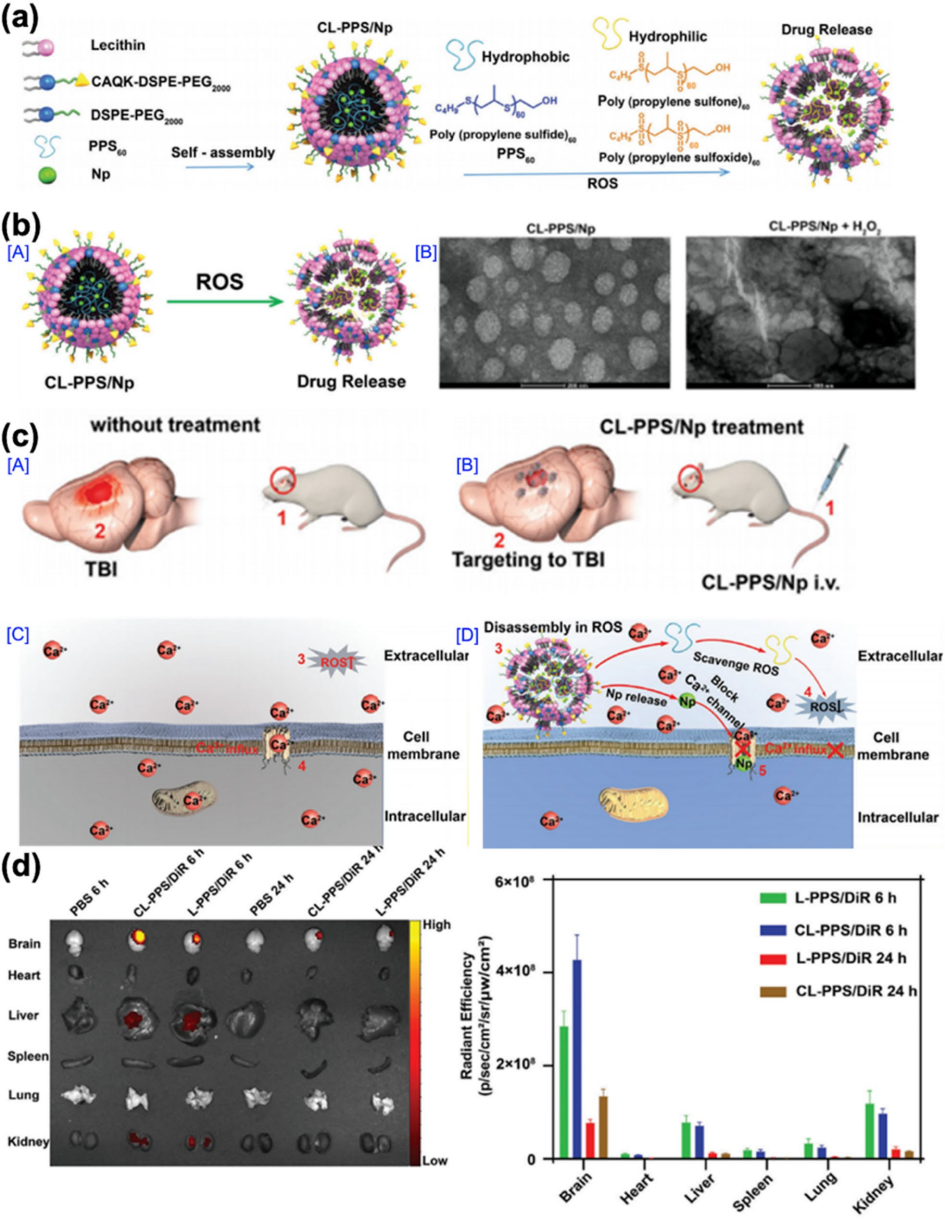
DSPE-based LNPs for TBI treatment. (**a**) Schematic of the ROS-triggered CL-PPS/Np drug delivery system. The formation and mechanism action of the CL-PPS/Np formulation. CL-PPS/Np comprises an outer lipid surface (lecithin, CAQK-DSPE-PEG_2000_, and DSPE-PEG_2000_) and an inner PPS polymer core that entraps hydrophobic Np. The DSPE-PEG_2000_ coating on the PPS polymer significantly increased plasma circulation, while CAQK-DSPE-PEG_2000_ enhanced TBI-target ability. In the high-ROS environment produced post-TBI, the hydrophobic PPS_60_ was oxidized to hydrophilic poly(propylene sulfone)_60_ and poly(propylene sulfoxide)_60_, leading to disassembly of the CL-PPS/Np structure and subsequent release of Np. (**b**) Schematic diagram of ROS-responsive release of Np from CL-PPS/Np in a high-ROS environment. TEM images of CL-PPS/Np and CL-PPS/Np + H_2_O_2_. (**c**) Schematic illustration of the therapeutic mechanism. In untreated TBIs, elevated ROS levels lead to Ca^2+^ influx and subsequent neuronal cell death. In contrast, treatment with CL-PPS/Np in TBI model mice reduced neuronal apoptosis and neuroinflammation by targeting the injury site and responding to the oxidative environment. On the other hand, upon administration in TBI model mice, CL-PPS/Np modified with CAQK selectively targeted the injury site. In the high ROS environment, CL-PPS/Np underwent oxidative degradation, releasing Np. Hydrophobic PPS_60_ was oxidized to hydrophilic poly(propylene sulfone)_60_ and poly(propylene sulfoxide)_60_ in the high ROS environment, effectively scavenging extracellular ROS. The released Np blocked L-type Ca^2+^ channels, and co-treatment of Np and PPS_60_ reduced neuronal cell death and neuroinflammation. (**d**) Fluorescent images of tissue distribution in TBI mice intravenously injected with L-PPS/DiR, CL-PPS/DiR, and PBS, along with quantification of DiR radiant efficiency in various tissues 6 and 24 h after a TBI (*n* = 5) [70]. Copyright (2022) John Wiley and Sons

In the fifth example, Liu and co-workers developed CAQK peptide-functionalized, ROS-responsive LNPs (CL-PPS/Np) to prevent secondary brain injury following a TBI. The design was based on the dual contribution of oxidative stress and calcium (Ca²⁺) overload in exacerbating neuronal damage post-injury. The authors hypothesized that a nano-platform capable of scavenging ROS and inhibiting intracellular Ca²⁺ influx could mitigate these secondary injury mechanisms. CL-PPS/Np consisted of a lipid-coated PPS polymeric core, composed of lecithin, CAQK-DSPE-PEG, and DSPE-PEG2000 (Fig. 10a). The hydrophobic PPS core was sensitive to ROS and transformed into a hydrophilic form upon oxidation, leading to nanoparticle disassembly and controlled drug release (Fig. 10b). This design enabled targeted accumulation at the TBI injury region via CAQK-mediated homing and ROS-triggered release the encapsulated agent, which in turn blocked L-type Ca²⁺ channels and eliminated extracellular ROS, thereby reducing neuronal apoptosis and neuroinflammation (Fig. 10c). In vivo biodistribution analysis confirmed that selective accumulation of CL-PPS/Np at the TBI site, with the highest signal intensity observed at 6 h post-injection (Fig. 10d). These findings demonstrated the therapeutic potential of functionalized LNPs in mitigating secondary injury by modulating oxidative stress and inhibiting calcium overload in TBIs.

In the sixth example, Kwon and co-workers investigated the impact of the PEG-lipid anchor length on the pharmacokinetics and brain accumulation of LNPs in a TBI mouse model. This study was based on the transient BBB disruption following a TBI that allows the passive accumulation of systemically administered nanoparticles. The authors hypothesized that modulation of PEGylation density would alter LNP circulation time and enhance targeted accumulation at the injury site.

**Fig. 11:**
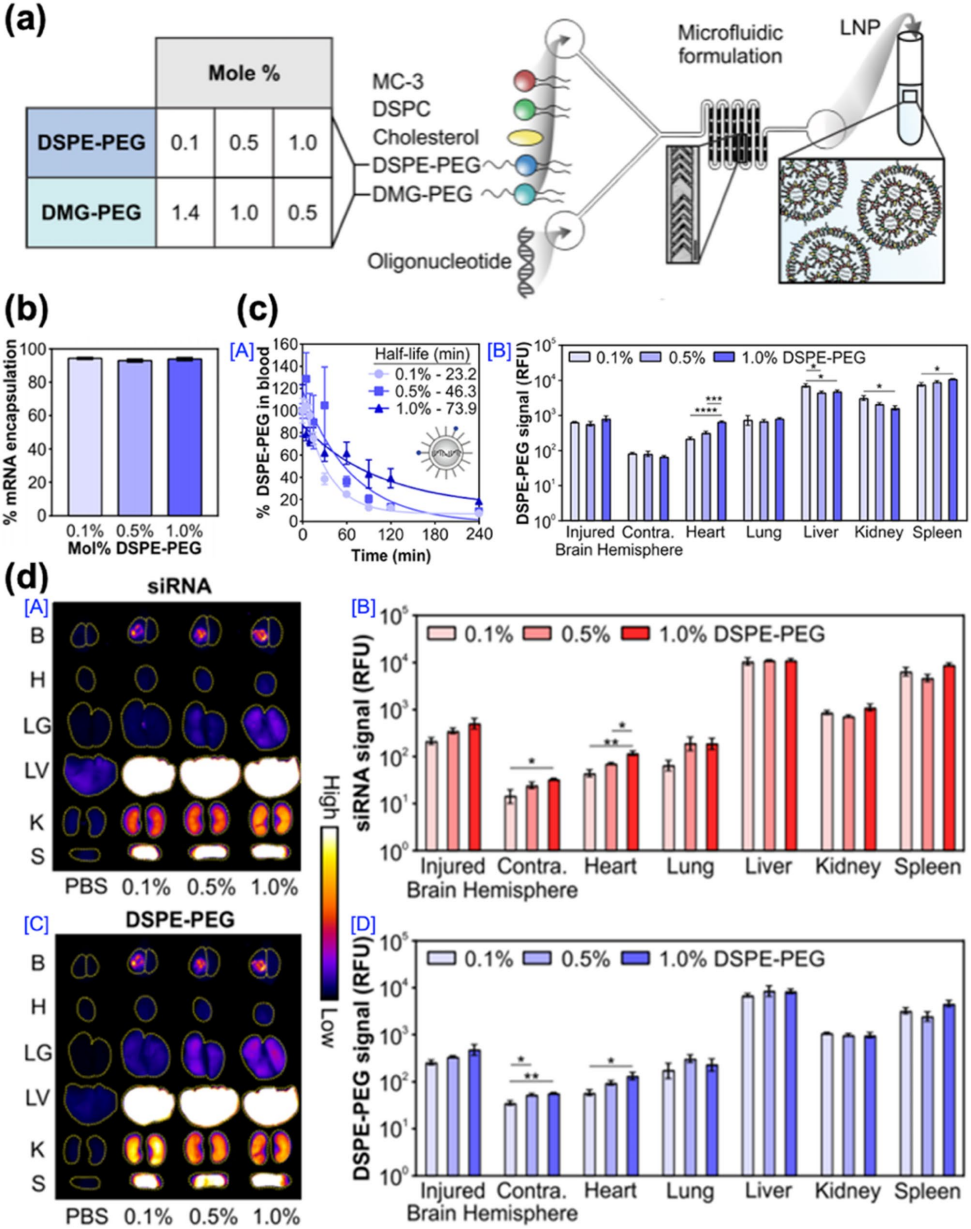
Therapeutic strategies utilizing lipid component-based nanoparticles for TBI treatment. (**a**) Formulation of LNPs with varying PEG-lipid compositions. **(b)** Encapsulation efficiency of mRNA within LNPs (*n* = 2). (**c**) Pharmacokinetic profiles of mRNA-loaded LNPs in a mouse TBI model as a function of PEG-lipid composition. [A] Blood circulation half-life and [B] organ biodistribution of mRNA LNPs labeled with Cy7-DSPE-PEG (*n* = 3; mean ± SEM; one-way ANOVA with Tukey’s post-test; *p < 0.05, ***p < 0.001, ****p < 0.0001). (**d**) Representative fluorescent images of whole organs and quantitative analysis of LNP accumulation [A, B] siRNA and [C, D] DSPE-PEG. Brains were analyzed by integrating the signal from the injured and uninjured contralateral hemispheres (*n* = 3; mean ± SEM; one-way ANOVA with Tukey’s post-test; *p < 0.05, **p < 0.01) [117]. Copyright (2023) Royal Society of Chemistry

LNPs were formulated using MC-3, DSPC, cholesterol, DMG-PEG, and varying ratios (0.1%, 0.5%, 1.0%) of DSPE-PEG (Fig. 11a). DLS analysis confirmed uniform particle sizes across all formulations. Despite differences in PEG density, mRNA encapsulation efficiency remained consistently high (Fig. 11b). Pharmacokinetic studies revealed that increasing the DSPE-PEG content prolonged the half-life of LNPs from 23.2 min (0.1%) to 73.9 min (1.0%) (Fig. 11c [A]). In vivo biodistribution analysis showed enhanced accumulation of LNPs in the injured hemisphere of the brain with increasing DSPE-PEG content, while reducing off-target distribution to peripheral organs (Fig. 11c [B], 11d). These results highlight the importance of PEG-lipid composition in optimizing the pharmacokinetics and delivery efficiency of LNPs in TBI treatment.

Collectively, these two studies illustrate the versatility of LNPs as nanotherapeutic platforms for TBIs. By integrating targeting peptides, ROS-responsive cores, and modulation of PEGylation density, LNPs can be engineered to effectively address major pathological features of TBIs, including oxidative stress, calcium overload, and BBB disruption. These strategies lay a solid foundation for the development of clinically translatable LNP-based nanotherapeutics to improve neuroprotection and functional recovery in TBI patients. Additionally, these metrics enable precise evaluation of LNP activity in the TBI model and highlight critical elements for the development of LNP-based therapeutics that selectively target injured brain regions. These studies suggest that mRNA could be utilized as a therapeutic agent and that the efficacy of LNPs can be optimized by adjusting anchor length to overcome TBIs.

### siRNA-based nanoparticles

**Fig. 12:**
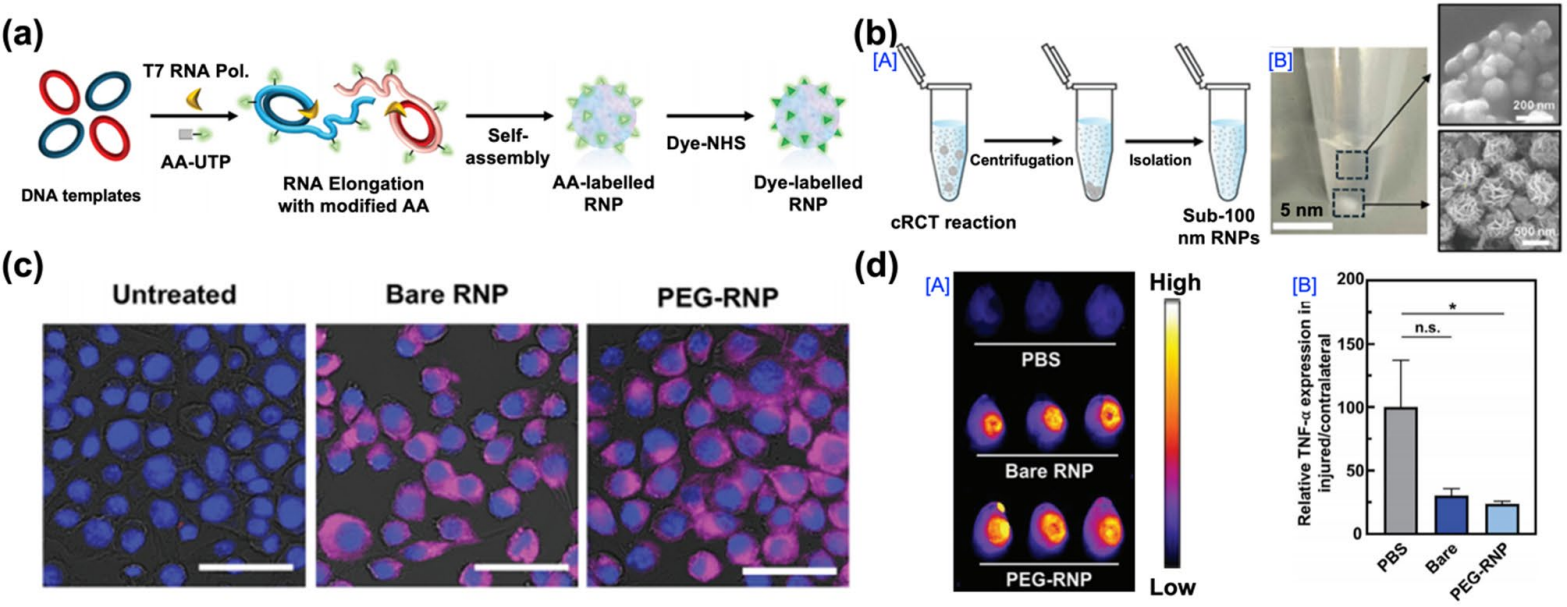
DNA/RNA-based nanoparticles for therapeutic strategies in TBI treatment. (**a**) Schematic illustration of DNA templates showing aminoallyl (AA) incorporation into RNA nanoparticles (RNPs), followed by NHS-fluorophore dye to produce dye-labeled RNPs. (**b**) [A] Schematic illustration of sub-100 nm RNP synthesis via cRCT and subsequent isolation via centrifugation. [B] Image of the cRCT reaction after centrifugation and SEM images of RNA particles isolated from both the supernatant and precipitate. (**c**) Fluorescence microscopy images of Cy5-labeled bare RNPs and PEG-RNPs (magenta) in BV2 cells after 24 h. Scale bar = 50 μm. (**d**) [A] Fluorescence images showing RNPs accumulation in the injured right hemisphere of the brain. [B] Quantitative comparison of bare RNPs and PEGylated RNPs in the brain [118]. Copyright (2024) John Wiley and Sons

The seventh example is a siRNA-based nanoparticle system developed by Peixuan Guo and co-workers in 2024 (Fig. 12). This study introduced PEGylated multimeric RNA nanoparticles designed for systemic delivery of siRNA to the injured brain following a TBI. The design was based on the need for effective gene-silencing therapies to regulate secondary injury mechanisms, which are primarily driven by the overexpression of inflammatory cytokines and neurotoxic mediators. These molecular events contribute to progressive neuronal damage, yet no approved therapies currently exist that improve long-term brain health. RNA interference (RNAi) therapy offers a promising strategy by suppressing detrimental gene expression, such as that of pro-inflammatory cytokines. However, RNA-based therapeutics face significant challenges, including limited BBB permeability and poor in vivo stability, which restrict their practical application. To address these challenges, the authors designed multimeric RNA nanoparticles with PEGylated surfaces to improve stability, prolong circulation time, and promote accumulation at the injury site. The RNA nanoparticles were synthesized using a rolling circle transcription (RCT) method, which enabled large-scale production of multimeric RNA strands, followed by self-assembly into three-way junction (3WJ) nanostructures and surface PEGylation (Fig. 12a). A dye-labeling process was incorporated to facilitate imaging of in vivo biodistribution. The sub-100 nm nanoparticles were purified through centrifugation and isolation steps, and TEM imaging confirmed their uniform, spherical morphology (Fig. 12b). In vitro studies demonstrated that PEGylated RNA nanoparticles exhibited high stability and low cytotoxicity. Cellular uptake analysis in BV2 microglial cells revealed efficient internalization of PEGylated RNA nanoparticles, which was superior to that of bare RNA nanoparticles (Fig. 12c). Furthermore, the RNA nanoparticles were designed to carry siRNA sequences targeting TNF-α, a key proinflammatory cytokine implicated in TBI pathology. Following systemic administration in a murine CCI model, in vivo fluorescence imaging revealed that PEGylated RNA nanoparticles preferentially accumulated in the injured hemisphere of the brain, with minimal off-target distribution (Fig. 12d [A]). Quantitative analysis further confirmed significant downregulation of TNF-α expression in the injured brain after treatment with PEGylated RNA nanoparticles, compared to both untreated controls and bare RNA nanoparticle-treated groups (Fig. 12d [B]). This study provides compelling evidence that PEGylated multimeric RNA nanoparticles can effectively deliver siRNA to the injured brain and achieve gene silencing therapy in TBIs.

The use of rolling circle transcription technology enabled the scalable fabrication of RNA nanoparticles. Their small particle sizes, high serum stability, efficient gene knockdown, and targeted accumulation highlight the potential of this platform as a clinically translatable nanotherapeutic strategy. These findings suggest that siRNA-based nanoparticles offer a promising approach for modulating gene expression and attenuating secondary injury in TBI treatment.

**Table 2:** Nanosensors for TBIs with key features and therapeutic strategies

Chapter	Materials	Key features & nanosensing strategy	Applications
4.1	Peptide-based nanosensors	Calpain-1 activity-responsive peptide nanosensor with PEG scaffold for activity-based TBI diagnosis.	Real-time detection of calpain-1 activity for early-stage TBI diagnosis.
4.2	ECM-targeted nanosensors	ECM-targeted nanosensor modified with HA-binding peptides to enhance accumulation and cleavage by calpain-1.	Enhanced accumulation and sensitivity of nanosensors at the injured ECM in TBI.
4.3	Biomarker-responsive nanosensors	Activity-based nanosensor releasing cleaved peptide biomarker into biofluids for minimally invasive detection.	Minimally invasive measurement of protease activity through blood or urine analysis.
4.4	Polymer-based nanosensors	Polymeric nanosensor combining CAST and FRET substrate peptide for activity sensing and inhibition.	Simultaneous detection and inhibition of calpain activity to mitigate secondary injury in TBI.
4.5	Fibrinogen-based nanosensors	Fibrinogen-modified nanosensor using click chemistry for selective localization at fibrin clots in TBI site.	Targeted localization of therapeutic and diagnostic agents at TBI-associated clots.

ECM: extracellular matrix HA: Hyaluronic acid. CAST: Calpain inhibitory peptide. FRET: Fluorescence resonance energy transfer

## Nanosensors for TBI

This chapter provides a summary of the nanomaterials-based sensing approaches for monitoring TBI-related biomarkers as diagnostic tools. The chapters are organized to consider the clinical aspects of the advanced strategy, including the ability to distinguish between specifically damaged and healthy tissue after primary injury using nanosensors, noninvasive real-time monitoring of brain injury pathology, and personalized diagnosis and treatment. It consists of five sections: (1) Peptide-based nanosensors (2) ECM-targeted nanosensors, (3) Biomarker-responsive nanosensors, (4) Polymer-based nanosensors, and (5) Fibrinogen-based nanosensors (Table 2).

### Peptide-based nanosensors

**Fig. 13:**
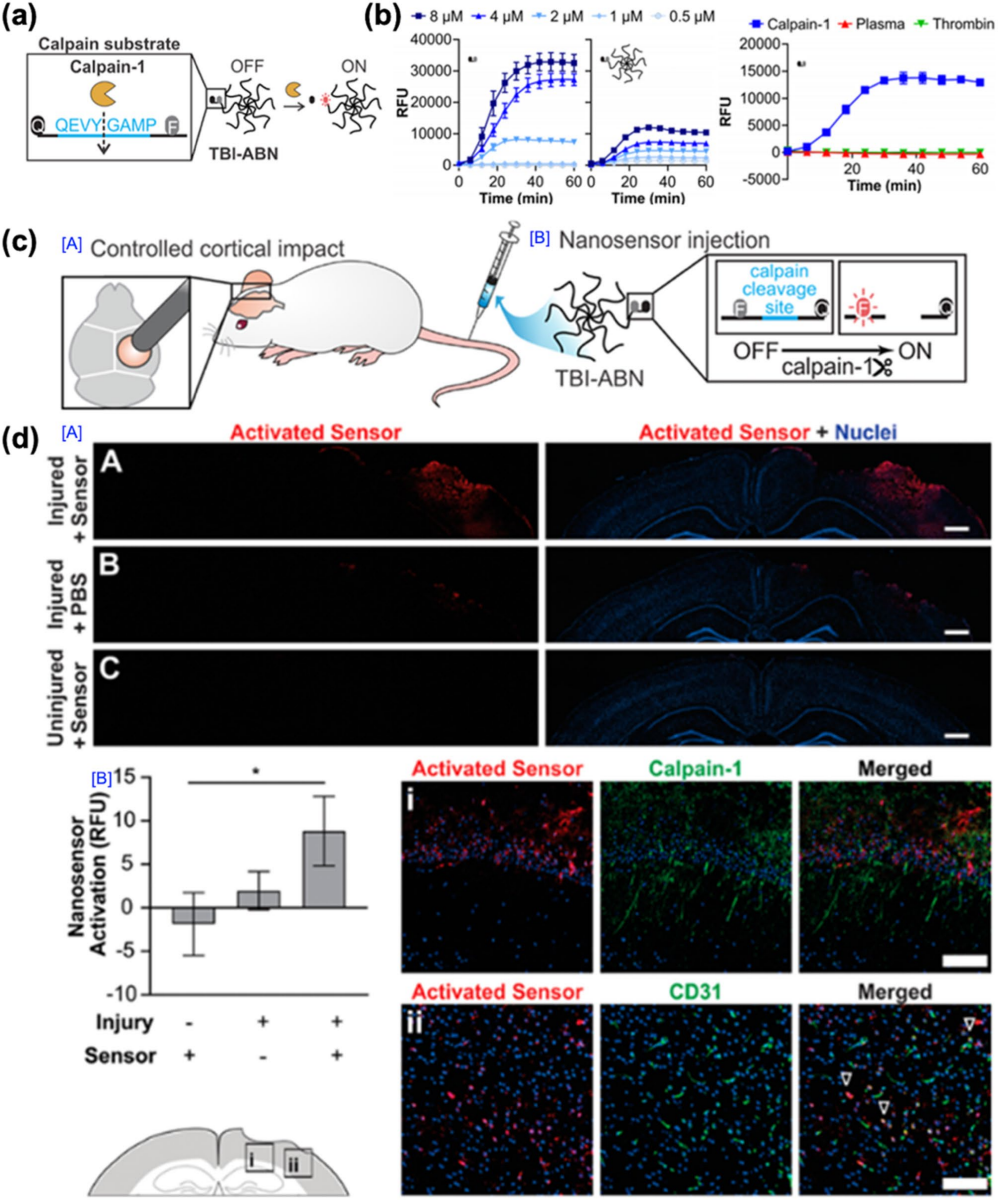
Peptide-based nanosensors for TBI diagnosis. (**a**) Schematic diagram of the development of TBI-ABN. (**b**) Comparison of the peptide (before cleavage) and PEG (after cleavage) in terms of dosage, with the ratio of peptide to PEG being 1:1. (**c**) Schematic of in vivo TBI modeling and the treatment of TBI-ABN, selectively regulated by calpain-1 activity. (**d**) Activation of the calpain sensor in the injured brain tissue following intravenous delivery, with representative coronal brain slices from both injured and uninjured brains for comparison (blue: nuclei; red: activated nanosensor, scale bar = 500 μm). Quantification of the mean sensor intensity in the injured hemisphere, normalized to the uninjured control brains, was performed. Slices showing sensor localization relative to calpain-1 and CD31 in the injury periphery (blue: nuclei; red: activated nanosensor; green: calpain-1 or CD31; outlined arrows: the overlap of the sensor with CD31, scale bar = 100 μm) [58]. Copyright (2020) American Chemical Society

In the first example, reported in 2020 by Ester J. Kwon and co-workers, a peptide-based nanosensor strategy was introduced for real-time diagnosis and monitoring of TBIs (Fig. 13). This nanosensor, termed the traumatic brain injury activity-based nanosensor (TBI-ABN), was designed to detect protease activity associated with neuronal injury. The design was based on the pathological feature that calpain-1, a calcium-sensitive protease, is overactivated following a TBI and contributes to cytoskeletal degradation and neuronal apoptosis.

Conventional diagnostic methods, such as computed tomography (CT) and magnetic resonance imaging (MRI), are limited in their ability to detect dynamic biochemical changes associated with TBIs and often lack the sensitivity needed to monitor injury progression. To address these limitations, the authors hypothesized that an activity-based nanosensor capable of detecting calpain-1 activity could provide a sensitive and minimally invasive diagnostic tool. TBI-ABN was synthesized by conjugating a fluorogenic calpain-1 substrate peptide (QEVYIGAMP) to an 8-arm PEG scaffold, forming a FRET-based nanosensor (Fig. 13a). The molecular weight of the PEG carrier was optimized to 40 kDa to facilitate prolonged circulation, passive accumulation at the injury site through transient BBB disruption, and minimal renal clearance. In vitro studies demonstrated that the peptide substrate was selectively cleaved by recombinant calpain-1, with negligible response to plasma proteases or thrombin (Fig. 13b). Additionally, the nanosensor retained its protease responsiveness after conjugation, although cleavage efficiency was slightly reduced due to steric hindrance.

In vivo experiments were conducted using a CCI mouse model. Following systemic administration, TBI-ABN accumulated at the injured brain site and was selectively activated by calpain-1 activity. Fluorescence imaging of coronal brain sections confirmed signal localization at the injury site, with minimal activation observed in uninjured or sham-injured mice (Fig. 13c). Immunofluorescence analysis further validated the spatial co-localization of sensor activation with calpain-1 and the vascular marker CD31, demonstrating the nanosensor’s ability to penetrate the BBB and detect injury-associated enzymatic activity (Fig. 13d).

This study provides compelling evidence that peptide-based nanosensors utilizing activity-dependent fluorescence activation offer a minimally invasive, real-time platform for diagnosing and monitoring TBIs. The high specificity of the nanosensor for calpain-1 and its ability to distinguish between injured and healthy tissue underscores its potential for advancing TBI diagnostics and guiding clinical decision-making.

### ECM-targeted nanosensors

**Fig. 14:**
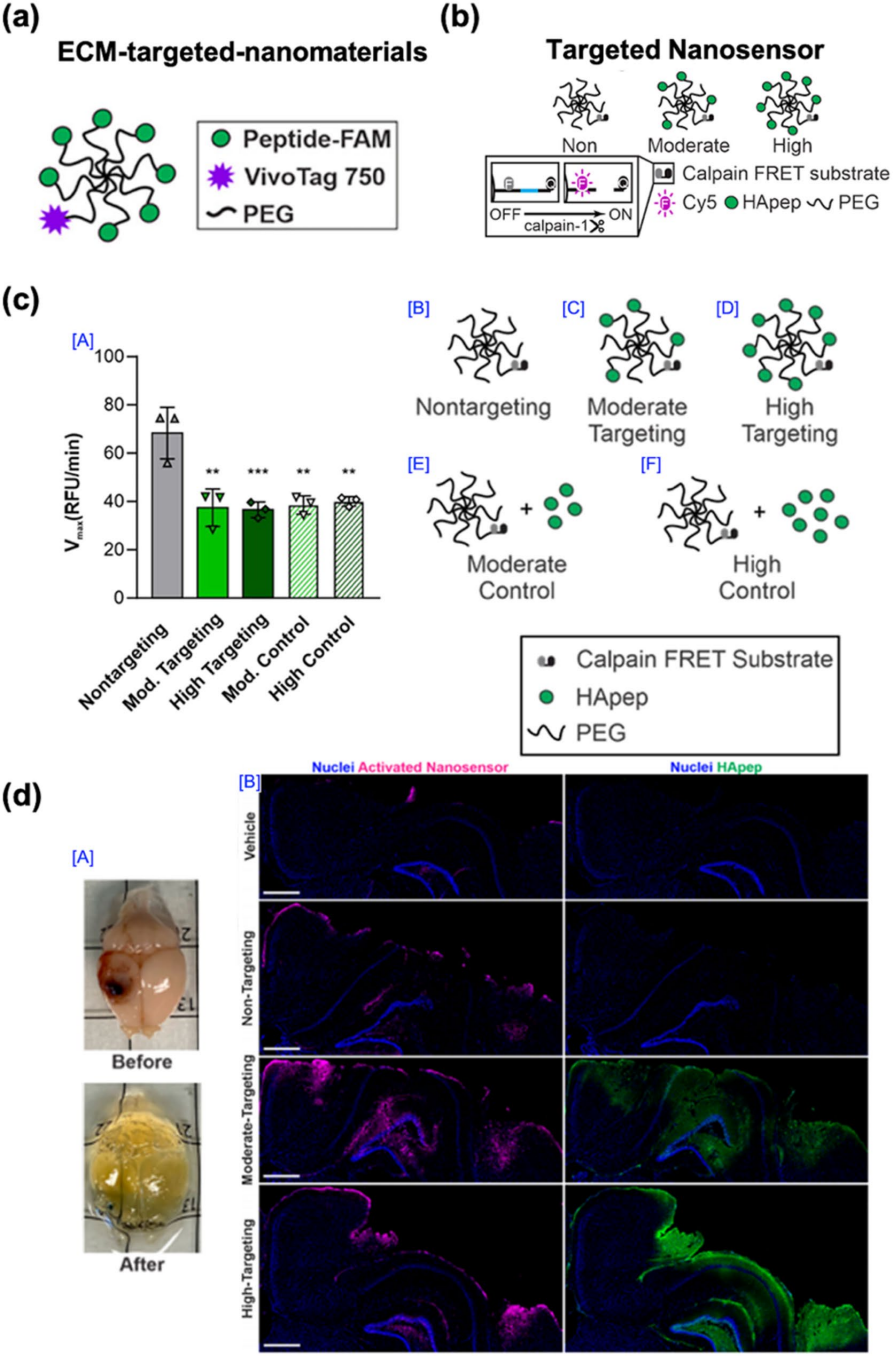
ECM-targeted nanosensors characterization of a TBI. (**a**) Illustration of ECM-targeted nanomaterials. (**b**) Schematic of hyaluronic acid (HA) pep-modified nanosensors with nontargeting, moderate, and high targeting capabilities. (**c**) Maximal cleavage velocities of TBI-ABNs (8 µM), quantified by calpain FRET substrate peptide, were assessed after incubation with human calpain-1 and varying levels of conjugated HApep (for targeting conditions) or unconjugated HApep (for control conditions) (*n* = 3; mean ± SD; **p < 0.01, *p < 0.001; one-way ANOVA with Tukey’s post-hoc test). (**d**) Images of the injured brain before and after whole brain CUBIC clearing and the overall TBI-ABN distribution within coronal sections of the injured hemisphere in CCI-injured mice [58]. Copyright (2021) American Chemical Society

In the second example, reported in 2021 by Ester J. Kwon and co-workers, an ECM-targeted nanosensor strategy was developed to enhance signal generation and localization of activity-based nanosensors for TBI diagnosis (Fig. 14). This study built upon their previous work on peptide-based nanosensors by incorporating an ECM-targeting motif to improve sensor accumulation at the injury site. The design was based on the pathological observation that TBIs induce significant ECM remodeling in the injured brain, increasing fibronectin expression at the injury periphery. Traditional nanosensor designs primarily relied on passive accumulation in injured brain regions through transient BBB disruption. However, this passive strategy had inherent limitations in targeting efficiency and signal intensity. To address this issue, the authors hypothesized that incorporating an ECM-binding peptide into the nanosensor design would enhance site-specific localization and signal activation.

The ECM-targeted nanosensor was synthesized by modifying the previously developed TBI-ABN with a fibronectin-binding peptide (FBC) conjugated to an 8-arm PEG scaffold (Fig. 14a). This dual-functional platform enabled the nanosensor to recognize injury-associated ECM components, while also responding to calpain-1 activity via fluorescence activation. In vitro studies demonstrated that the fibronectin-binding capacity of the modified nanosensor was retained without compromising protease responsiveness.

Cleavage kinetics assays confirmed that the targeted nanosensor was selectively cleaved by calpain-1, and the cleavage velocity was evaluated under varying degrees of peptide modification (Fig. 14b and c). In vivo experiments using a CCI mouse model indicated that, following systemic administration, the ECM-targeted nanosensor exhibited enhanced accumulation and fluorescence activation at the injury site compared to the non-targeted TBI-ABN, as visualized by CUBIC tissue clearing and optical imaging (Fig. 14d). Quantitative analysis revealed significantly higher sensor intensity in the injured cortex with moderate and high levels of ECM-targeting modification. This study demonstrated that ECM-targeted nanosensors can enhance the sensitivity and spatial precision of activity-based TBI diagnostics. By leveraging injury-induced ECM remodeling, specifically the fibronectin overexpression, the authors demonstrated that nanosensor localization and activation at the injury site were markedly enhanced, offering a promising strategy for non-invasive, real-time monitoring of TBI pathology.

### Biomarker-responsive nanosensors

**Fig. 15:**
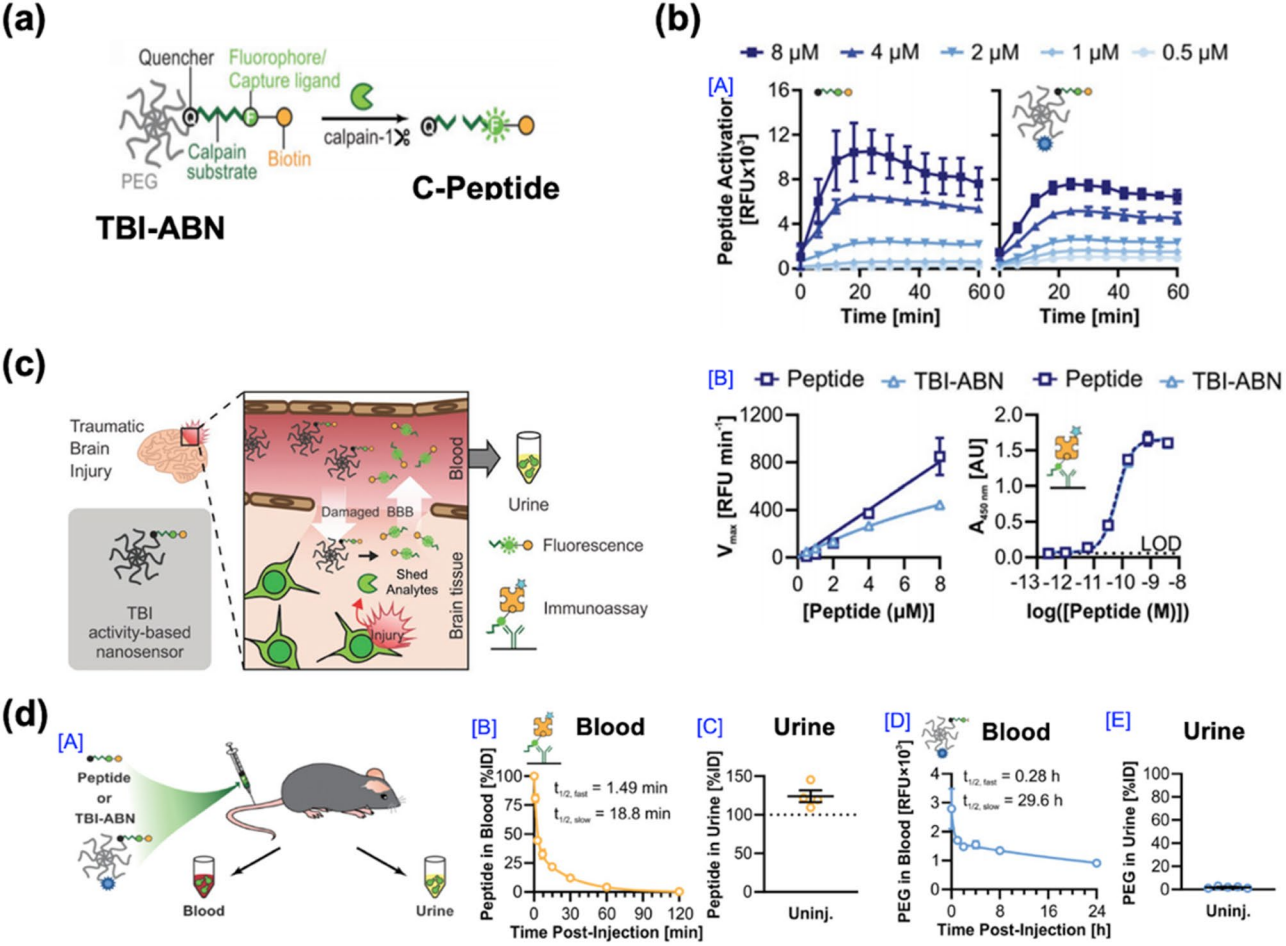
Utilization of biomarker-responsive peptide formulation for TBI diagnosis. (**a**) Illustration of TBI-ABN, which releases a cleaved peptide (c-Peptide) biomarker after cleavage of the peptide substrate by calpain-1. (**b**) In vitro cleavage kinetics of free peptide (left) and peptide conjugated with VT750 and PEG to form TBI-ABN (right), incubated with human calpain-1. (**c**) Overview of diagnostic TBI-ABN. After systemic administration, ABN crosses the compromised BBB and accumulates in the injured brain tissue where it is cleaved by calpain-1. The resulting cleaved peptide (c-Peptide) was subsequently released into the bloodstream and urine, enabling minimally invasive measurement of protease activity via fluorescence or immunoassay. (**d**) Pharmacokinetics of TBI-ABN in mice. The nanosensor was administered intravenously to uninjured mice, with blood and urine samples collected at multiple time points post-injection. The half-life of the free calpain substrate in the blood circulation of uninjured mice was quantified by ELISA [43]. Copyright (2023) John Wiley and Sons

In the third example, reported in 2023 by Ester J. Kwon and co-workers, a biomarker-responsive nanosensor strategy was developed for the minimally invasive diagnosis of TBIs (Fig. 15). Conventional diagnostic methods, such as CT and MRI, have limited sensitivity in detecting mild TBIs and require expensive equipment, highlighting the need for alternative diagnostic platforms. To address this gap in clinical diagnostics, the research team designed an activity-based nanosensor (TBI-ABN) that utilizes calpain-1 activity, a TBI-associated protease, as a biomarker.

TBI-ABN consists of an 8-arm PEG scaffold conjugated with a FRET peptide substrate containing a calpain-1 cleavage site and a biotin moiety. Upon cleavage by calpain-1 in the injured brain, the cleaved peptide fragment (c-Peptide) is released and can be detected in biofluids such as blood and urine (Fig. 15a). In vitro kinetic analysis demonstrated that the peptide substrate was efficiently cleavaged by calpain-1, both in free form and when conjugated to the nanosensor (Fig. 15b). In vivo after systemic administration in a mouse model of a TBI, the nanosensor accumulated at the injured brain site, where increased calpain-1 activity triggered peptide cleavage. The released c-Peptide then crossed the damaged BBB and was detectable in the blood and urine, enabling a minimally invasive diagnostic approach (Fig. 15c). Furthermore, the pharmacokinetics of c-Peptide and TBI-ABN were evaluated in blood and urine samples, revealing that the released peptide exhibited favorable circulation and elimination profiles for diagnostic use (Fig. 15d). This study demonstrated that measuring calpain-1 activity using a nanosensor platform provides a sensitive and non-invasive method for TBI diagnosis, with promising potential for future clinical translation.

### Polymer-based nanosensors

**Fig. 16:**
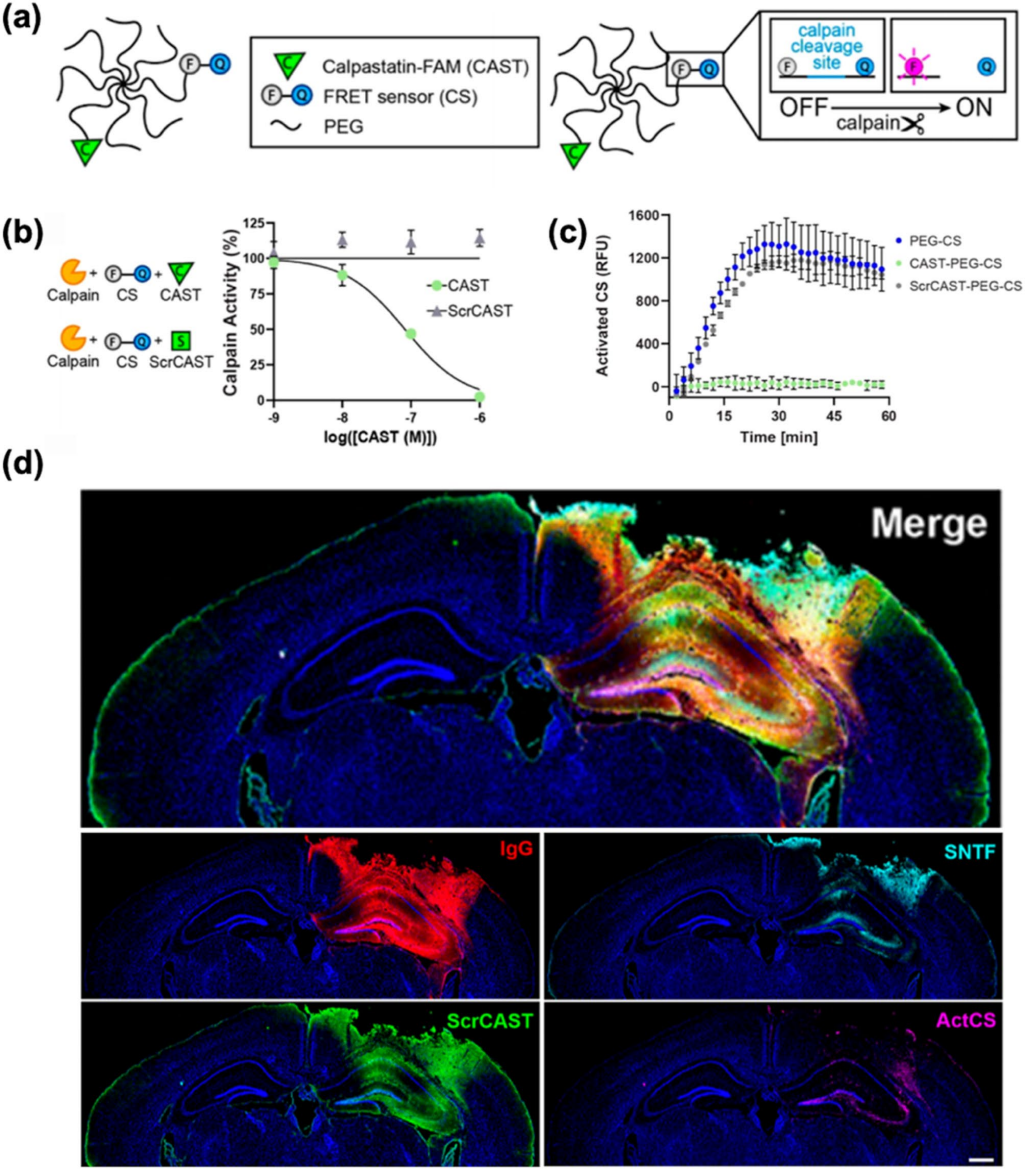
Polymeric nanosensors for monitoring TBI protease activity. (**a**) Schematic of the nanotheranostic composed of a PEG nanomaterial scaffold, calpastatin-FAM (CAST), calpain sensor (CS) peptide, and substrate peptide activation by calpain. (**b**) Titration of CAST for calpain-1 inhibition. (**c**) Activation of the CS peptide in the nanotheranostic materials mediated by recombinant human calpain-1. (**d**) Coronal brain sections of mice were administered with ScrCAST − PEG − CS. The sections were stained for endogenous IgG, FAM, and cleaved spectrin (red: IgG; green: FAM-labeled nanomaterial; cyan: SNTF; magenta: activated CS peptide, scale bar = 500 μm) [69]. Copyright (2024) American Chemical Society

In the fourth example, reported in 2024 by Ester J. Kwon and co-workers, a polymer-based nanosensor strategy was developed to facilitate activity-based diagnosis of TBIs by targeting the calpain-1 activity (Fig. 16).

Conventional imaging modalities for TBI diagnosis often face limitations, including high cost, poor prognostic capability, and an inability to provide real-time molecular information. To overcome these challenges, the research team proposed an innovative polymeric nanosensor system designed to selectively detect calpain-1 activity in injured brain tissue. The design was based on the pathological role of calpain-1 in TBIs. Calpain-1, a calcium-sensitive cysteine protease, is known to degrade cytoskeletal and signaling proteins upon activation, contributing to neuronal injury and apoptosis. Monitoring calpain-1 activity can provide real-time insights into the severity and progression of TBIs. The nanosensor, referred to as CAST-PEG-CS, consists of a PEG-based polymeric scaffold conjugated with two functional peptides: (1) CAST (calpain inhibitory peptide) and (2) CS (FRET-based sensor peptide). The sensor operates through a FRET mechanism, where calpain-1 cleaves the CS peptide and a fluorescence signal is generated, while the CAST peptide simultaneously inhibits calpain-1 activity (Fig. 16a). In vitro analysis demonstrated that CAST-PEG-CS exhibited strong inhibition of calpain-1 activity, with a dose-dependent reduction in protease activity confirmed by FRET assay (Fig. 16b). Additionally, the kinetic analysis revealed that CAST-PEG-CS effectively suppressed nanosensor activation over time when compared to the control ScrCAST-PEG-CS (Fig. 16c).

In vivo evaluation in a TBI mouse model showed that ScrCAST-PEG-CS was widely distributed in the injured brain regions, as evidenced by histological analysis (Fig. 16d). This selective accumulation at the injury site was attributed to the nanosensor’s responsiveness to elevated calpain activity and the compromised vasculature following a TBI. Collectively, this study demonstrated that polymer-based nanosensors, such as CAST-PEG-CS, offer a novel platform for real-time, activity-based diagnosis of TBI. In addition to diagnostic potential, the calpain-inhibitory function of CAST suggests possible therapeutic applications in mitigating secondary neuronal damage. These findings underscore the promise of polymeric nanosensor systems in advancing personalized diagnostic and therapeutic strategies for TBIs.

### Fibrinogen-based nanosensors

**Fig. 17:**
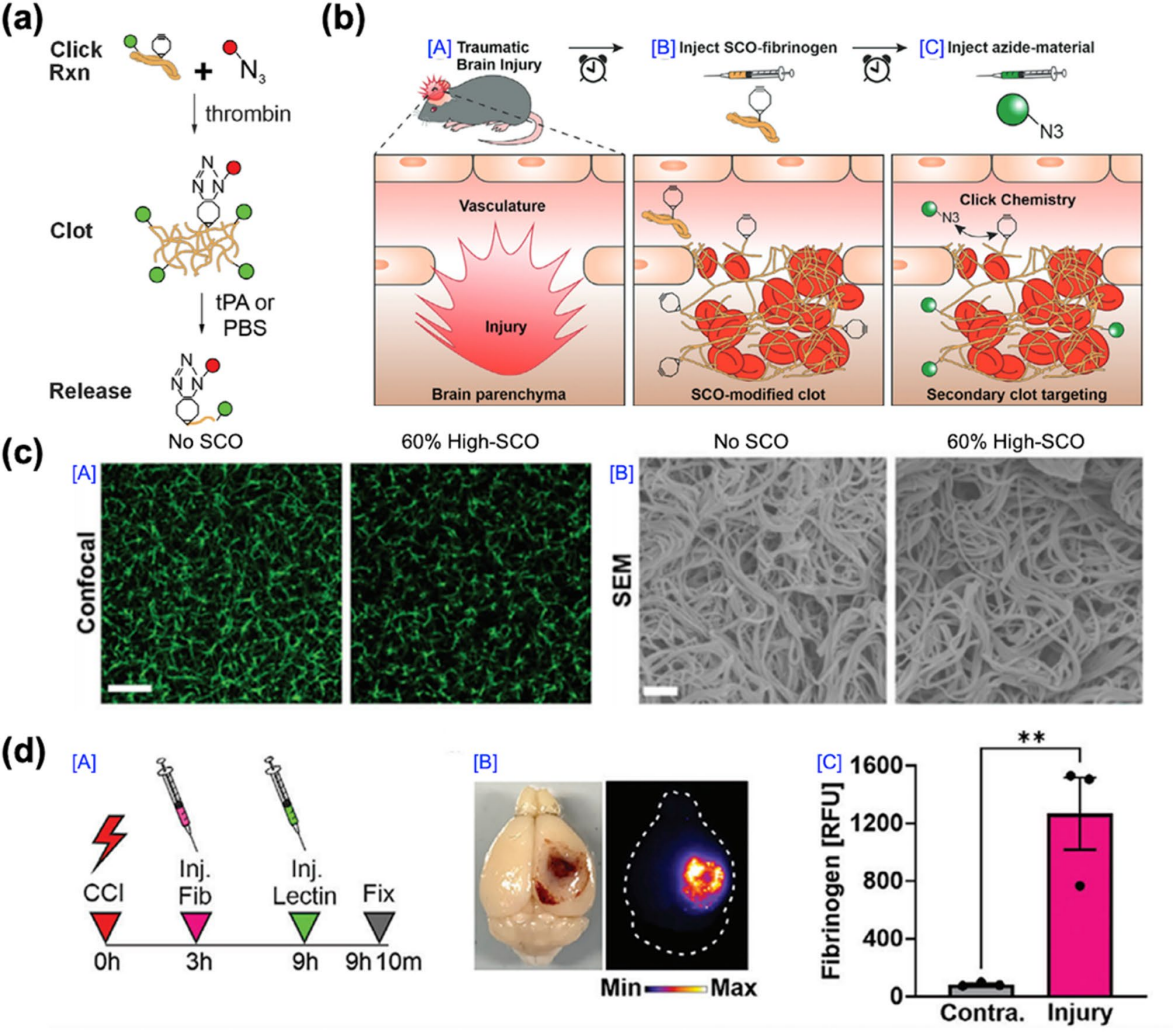
Application of click chemistry-based hybrid formulation for TBI fibrin clot. (**a**) Schematic of an in vitro assay for quantifying azide-dye release kinetics from SCO-fibrin clots. (**b**) Overview of the clot reprogramming strategy for the chemical capture of intravenously delivered materials. (**c**) Confocal and SEM images of representative samples: no SCO and 60 w/w% high SCO-modified fibrinogen labeled with AlexaFluor 488 (*n* = 3, Scale bar = 25 μm for confocal, 500 nm for SEM). (**d**) Fibrinogen is incorporated within clots in the injured brain. Schematic of dosing paradigm. Surface imaging displaying a fibrinogen signal colocalized with a visible clot in the injured cortex of the brain. Quantification of fibrinogen signal in the contralateral (contra.) and injury cortices (*n* = 3; mean ± SEM; **p < 0.01; unpaired two-tailed t-test) [119]. Copyright (2024) John Wiley and Sons

The fifth example, reported in 2024 by Ester J. Kwon and co-workers, describes the development of a fibrinogen-based nanosensor platform that leverages fibrin clot formation as a targeting strategy for TBI treatment (Fig. 17).

TBIs induce microvascular injury, leading to clot formation and disruption of the BBB, which presents challenges for effective drug delivery. The researchers hypothesized that fibrin clots, a pathological hallmark of a TBI, could serve as a scaffold for targeted delivery via click chemistry reactions. The design of this platform is based on the use of strained cyclooctene (SCO)-modified fibrinogen, which binds to fibrin clots at the injury site. Following systemic injection, the SCO-modified fibrinogen localized to the damaged vasculature, and subsequent administration of azide-functionalized therapeutic or diagnostic agents enabled covalent binding via click chemistry, thereby enhancing site-specific delivery (Fig. 17b). This strategy aims to overcome the limitations of non-specific accumulation and rapid clearance associated with conventional drug delivery systems. The clot-targeting system was synthesized by modifying fibrinogen with SCO moieties. Upon injury, the SCO-modified fibrinogen was intravenously delivered and deposited onto fibrin clots at the injury site. Azide-functionalized compounds were then administered, resulting in efficient chemical conjugation to the clot scaffold (Fig. 17a). Structural analysis by confocal microscopy and SEM confirmed that SCO-modified fibrinogen maintained its clot-forming capacity without impairing the clot structure (Fig. 17c).

In vivo, systemic administration of SCO-fibrinogen demonstrated preferential accumulation at the TBI site, with minimal signal observed in the contralateral hemisphere and no detectable signals in peripheral tissues. Quantitative analysis confirmed significantly higher fibrinogen signal intensity at the injury site, validating the clot-specific targeting capability of this nanosensor system (Fig. 17d). This study demonstrates that fibrinogen-based nanosensors, utilizing click chemistry, can achieve highly specific localization at TBI sites by targeting endogenous fibrin deposits. Since fibrin remains at the injury site for several days post-injury, this approach provides an extended therapeutic window for targeted delivery. The use of clot-targeting nanosensors offers a promising strategy to improve therapeutic efficacy and localization in TBIs, addressing critical barriers posed by vascular damage and BBB disruption.

## Conclusion and perspective

TBIs remain one of the most intricate and challenging neurological conditions due to the complexity of its secondary injury cascades, including oxidative stress, neuroinflammation, and BBB disruption. Despite extensive research efforts, effective clinical interventions are still limited. This review has outlined recent advances in theranostic nanomaterials engineered to overcome these challenges by integrating diagnostic and therapeutic functionalities into a single platform. Emerging nanotherapeutic systems, such as PEGylated nanoparticles, nano-sized polymers, targeting peptide conjugates, DNA/RNA-based constructs, lipid-based formulations, and nanozymes, have demonstrated enhanced capabilities for targeted drug delivery, neuroprotection, and suppression of neuroinflammation. Moreover, several single-platform systems have been designed to exploit the pathological microenvironment of TBI—such as elevated ROS levels, acidic pH, and abnormal GSH concentrations—for responsive drug release and imaging activation. These nanoplatforms offer significant advantages over conventional therapies by improving site-specific drug localization and minimizing systemic side effects. Concurrently, nanosensors have transformed the real-time diagnosis and monitoring of TBIs. Recent innovations, including ECM-targeted sensors, biomarker-responsive probes, and polymeric nanostructures, enable the dynamic detection of molecular changes associated with injury. These advanced systems facilitate the highly sensitive quantification of critical biomarkers, including calpain activity and its substrates. To overcome the complex pathophysiology of TBI, recent advances in nanotherapeutics and nanosensors have enabled more targeted and responsive intervention strategies. PEGylated nanozymes, MnO₂-based platforms, and metabolically active nanovesicles provide ROS scavenging, enhanced cerebral perfusion, and neuroprotective effects by leveraging the pathological microenvironment of TBI. In parallel, glutamate-sensitive electrochemical sensors, LDH-detecting microfluidic chips, and EIS-based systems offer real-time, noninvasive monitoring of neural damage. These multifunctional nanoplatforms highlight the potential of integrated theranostic approaches to improve both diagnosis and treatment outcomes in TBI.

Despite these advances, several scientific and translational barriers must still be addressed. First, the long-term biocompatibility, biodegradability, and clearance of nanomaterials remain critical concerns. While many systems have demonstrated efficacy in vitro and in vivo, comprehensive toxicological evaluations are required to assess potential risks of chronic exposure. Second, efficient and selective transport across the BBB remains a major obstacle. Although transient BBB openings can be utilized, achieving sustained and controlled delivery is still an unmet need. Third, the scalability, reproducibility, and regulatory approval of multifunctional nanoplatforms present significant hurdles. For clinical translation, standardized protocols for manufacturing and quality control must be established.

Looking forward, several strategic directions merit focused attention. Integrating artificial intelligence (AI) and machine learning into nanomaterial design may accelerate the optimization of therapeutic performance and enable personalized nanomedicine. AI-driven predictive modeling could guide the development of individualized nanoplatforms that maximize efficacy while minimizing off-target effects. In parallel, multifunctional systems capable of simultaneous drug delivery, imaging, and real-time monitoring will be pivotal in advancing TBI precision medicine. Furthermore, bioengineered carriers such as exosome-mimetic systems and cell membrane-coated nanoparticles may offer superior biocompatibility and immune evasion. The convergence of gene-editing technologies, particularly CRISPR-based modalities, with nanocarrier platforms, presents an exciting approach for precise modulation of TBI-associated genes, thereby promoting long-lasting neuroprotection and regeneration. Advances in organ-on-a-chip and bioprinting technologies also hold promise for developing physiologically relevant TBI models for preclinical screening. Equally important is the rational design of biodegradable nanomaterials with tunable degradation profiles, responsive to the TBI microenvironment (e.g., pH, enzymatic activity), aligning with therapeutic timelines and reducing chronic accumulation. Enhancing understanding of long-term toxicity and biodistribution is essential for ensuring safe clinical application. Innovations in biomimetic coatings and bioresponsive architectures could further mitigate adverse effects and improve therapeutic indices.

In summary, theranostic nanomaterials represent a transformative approach to TBI management, enabling the integration of diagnosis and therapy within a unified single system. Realizing their clinical potential will require continued interdisciplinary collaboration across nanotechnology, neuroscience, and translational medicine. By addressing existing limitations and harnessing emerging technological advances, the next generation of theranostic nanomaterials is well-positioned to redefine the diagnosis, treatment, and prognosis of TBIs.

## Supplementary Information

Supplementary Material 1.

Supplementary Material 2.

Supplementary Material 3.

Supplementary Material 4.

## Data Availability

No datasets were generated or analysed during the current study.
