# Supplementary figures and images for: Recent advances in theranostic nanomaterials for overcoming traumatic brain injury
Source: J Nanobiotechnology. 2025 Oct 29;23:692. doi: 10.1186/s12951-025-03685-4 (PMC12570421; doi:10.1186/s12951-025-03685-4)

**Graphical abstract**


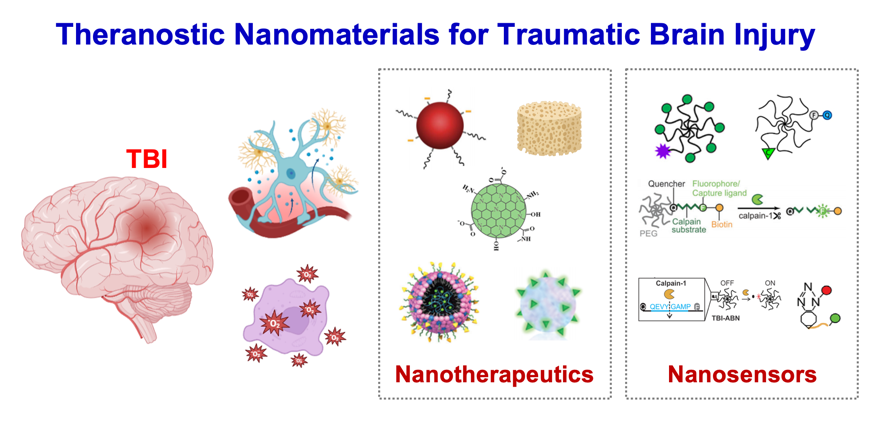

Supplement: Supplementary file 1 — Supplementary Material 1. [file 12951_2025_3685_MOESM1_ESM.docx]
