# Supplementary material for: Recent advances in theranostic nanomaterials for overcoming traumatic brain injury
Source: J Nanobiotechnology. 2025 Oct 29;23:692. doi: 10.1186/s12951-025-03685-4 (PMC12570421; doi:10.1186/s12951-025-03685-4)
Supplement: Supplementary file 2 — Supplementary Material 2. [file 12951_2025_3685_MOESM2_ESM.docx]

**Highlights**

- A comprehensive review of recent advancements in theranostic nanomaterials for traumatic brain injury (TBI) is provided.
- Key nanoplatforms are examined concerning material design, targeting strategies, and in vivo efficacy.
- Future perspectives on clinically translatable systems integrating diagnostic and therapeutic functionalities are presented.
