# Supplementary material for: Recent advances in theranostic nanomaterials for overcoming traumatic brain injury
Source: J Nanobiotechnology. 2025 Oct 29;23:692. doi: 10.1186/s12951-025-03685-4 (PMC12570421; doi:10.1186/s12951-025-03685-4)
Supplement: Supplementary file 3 — Supplementary Material 3. [file 12951_2025_3685_MOESM3_ESM.docx]

**Author Statement**

**Nam Cheol Hwang:** Writing – original draft, Writing – review and editing, Conceptualization, Investigation, Funding acquisition. **Dong Min Lim:** Writing – original draft, Writing – review and editing, Investigation. **Tae Sik Goh**: Writing – original draft. **Jung Mo Kang**: Writing – original draft. **Jaehoon Kim**: Writing – original draft. **Shin Kim**: Conceptualization, Investigation, Supervision, Funding acquisition, Writing – original draft, Writing – review and editing. **Yun Hak Kim**: Writing – original draft, Writing – review and editing, Conceptualization, Investigation, Supervision, Funding acquisition. **Dokyoung Kim:** Writing – original draft, Writing – review and editing, Conceptualization, Investigation, Supervision, Funding acquisition.
